# Systematic mapping of chemoreceptor specificities for *Pseudomonas aeruginosa*

**DOI:** 10.1128/mbio.02099-23

**Published:** 2023-10-04

**Authors:** Wenhao Xu, Jean Paul Cerna-Vargas, Ana Tajuelo, Andrea Lozano-Montoya, Melissa Kivoloka, Nicolas Krink, Elizabet Monteagudo-Cascales, Miguel A. Matilla, Tino Krell, Victor Sourjik

**Affiliations:** 1 Max Planck Institute for Terrestrial Microbiology & Center for Synthetic Microbiology (SYNMIKRO), Marburg, Germany; 2 Department of Biotechnology and Environmental Protection, Estación Experimental del Zaidín, Consejo Superior de Investigaciones Científicas, Granada, Spain; 3 Centro de Biotecnología y Genómica de Plantas CBGP, Universidad Politécnica de Madrid-Instituto Nacional de Investigación y Tecnología Agraria y Alimentaria/CSIC, Parque Científico y Tecnológico de la UPM, Pozuelo de Alarcón, Madrid, Spain; University of Wisconsin-Madison, Madison, Wisconsin, USA

**Keywords:** *Pseudomonas aeruginosa*, chemotaxis, signal transduction, ligand binding domains, chemoeffectors, receptor chimera, Förster resonance energy transfer (FRET), purines, amino acids, biogenic amines

## Abstract

The chemotaxis network, one of the most prominent prokaryotic sensory systems, is present in most motile bacteria and archaea. Although the conserved signaling core of this network is well characterized, ligand specificities of a large majority of diverse chemoreceptors encoded in bacterial genomes remain unknown. Here, we performed a systematic identification and characterization of new chemoeffectors for the opportunistic pathogen *Pseudomonas aeruginosa*, which has 26 chemoreceptors possessing most of the common types of ligand binding domains. By performing capillary chemotaxis assays for a library of growth-promoting compounds, we first identified a number of novel chemoattractants of varying strength. We subsequently mapped specificities of these ligands by performing Förster resonance energy transfer and microfluidic measurements for 16 hybrid chemoreceptors that combine the periplasmic ligand binding domains of *P. aeruginosa* receptors and the cytoplasmic signaling domain of the *Escherichia coli* Tar receptor. Direct binding of putative ligands to chemoreceptors was further confirmed using thermal shift assay and microcalorimetry. Altogether, the combination of methods enabled us to assign several new attractants, including methyl 4-aminobutyrate, 5-aminovalerate, L-ornithine, 2-phenylethylamine, and tyramine, to previously characterized chemoreceptors and to annotate a novel purine-specific receptor PctP. Responses of hybrid receptors to changes in pH further revealed a complex bidirectional pH sensing mechanism in *P. aeruginosa*, which involves at least four chemoreceptors PctA, PctC, TlpQ, and PctP. Our screening strategy could be applied for the systematic characterization of unknown sensory domains in a wide range of bacterial species.

**IMPORTANCE:**

Chemotaxis of motile bacteria has multiple physiological functions. It enables bacteria to locate optimal ecological niches, mediates collective behaviors, and can play an important role in infection. These multiple functions largely depend on ligand specificities of chemoreceptors, and the number and identities of chemoreceptors show high diversity between organisms. Similar diversity is observed for the spectra of chemoeffectors, which include not only chemicals of high metabolic value but also bacterial, plant, and animal signaling molecules. However, the systematic identification of chemoeffectors and their mapping to specific chemoreceptors remains a challenge. Here, we combined several *in vivo* and *in vitro* approaches to establish a systematic screening strategy for the identification of receptor ligands and we applied it to identify a number of new physiologically relevant chemoeffectors for the important opportunistic human pathogen *P. aeruginosa*. This strategy can be equally applicable to map specificities of sensory domains from a wide variety of receptor types and bacteria.

## INTRODUCTION

Most bacteria have evolved the ability to detect a wide range of environmental signals to survive and grow under rapidly changing conditions. One of the most prominent prokaryotic sensory systems is the chemotaxis network that controls motility (1, 2). Chemotaxis has multiple important functions in bacterial physiology, dependent on the lifestyle and ecological niche, enabling bacteria to accumulate toward optimal growth environments but also mediating collective behaviors and interactions with eukaryotic hosts (3, 4).

Such variety of chemotaxis-mediated functions is primarily ensured by the diversity of bacterial chemoreceptors, also called methyl-accepting chemotaxis proteins (MCPs) (5). While the core of the signaling pathway is conserved among bacteria, the number and specificity of chemoreceptors are highly variable and strain specific (6). The reported repertoire of signals recognized by chemoreceptors across bacterial species includes not only proteinogenic amino acids (7, 8), polyamines (9), quaternary amines (10), nucleobases and their derivatives (11, 12), organic acids (13, 14), and sugars (15) but also inorganic ions (16, 17), pH (18 – 20), and temperature (21, 22). Nevertheless, the signal specificity remains unknown for the absolute majority of chemoreceptors.

The paradigmatic model system of *Escherichia coli* chemotaxis consists of a single pathway, controlled by four transmembrane chemoreceptors and one aerotaxis receptor, and it includes six cytoplasmic signaling proteins: a histidine kinase CheA, an adaptor CheW, a response regulator CheY, a methyltransferase CheR, a methylesterase CheB, and a phosphatase CheZ (2). Typically, chemotactic stimuli modulate the autophosphorylation activity of CheA, which is inhibited by attractants and stimulated by repellents, subsequently altering the transphosphorylation of CheY. The phosphorylated CheY binds to the flagellar motor resulting in a change in the direction of flagellar rotation, ultimately causing a chemotactic response. CheZ is responsible for the dephosphorylation of CheY. After the initial pathway response, an adaptation system composed of CheR and CheB adjusts the level of receptor methylation on several specific glutamyl residues, providing negative feedback to the kinase activity, which ensures the adaptation of cells to persisting stimulation. Although most of the chemotaxis proteins found in *E. coli* are conserved across bacterial chemotaxis pathways, most bacteria have more complex chemosensory pathways, possessing additional chemotaxis proteins and chemoreceptors, alternative adaptation and signal termination strategies (23 – 25).

Canonical chemoreceptors can be separated in three functional domains: a periplasmic ligand binding domain (LBD), a signal conversion HAMP domain, and a cytoplasmic signaling domain that interacts with the autokinase CheA (5). Analyses of sequenced bacterial genomes revealed that bacterial chemoreceptors employ more than 80 different types of LBDs (6). In contrast, all *E. coli* transmembrane chemoreceptors possess the same four-helix bundle (4HB) type of LBD. Thus, although the *E. coli* chemotaxis signaling pathway is one of the simplest and best understood, it does not represent the diversity of bacterial sensory capabilities.

*Pseudomonas aeruginosa* is among the most important human pathogens, causing the death of more than half a million people annually, and it is also one of the most well-studied alternative models for chemotaxis (26, 27). The chemoreceptor repertoire of the *P. aeruginosa* model strain PAO1 has 26 chemoreceptors containing 12 different LBD types that feed into four different chemosensory pathways, of which 23 chemoreceptors were predicted to stimulate the genuine F6-type chemotaxis pathway that controls swimming motility. The other three receptors McpB/Aer2, WspA, and PilJ are involved in an F7-type pathway of unknown function, an alternative cellular function pathway that mediates c-di-GMP synthesis, and a type IV pili chemosensory pathway that is associated with twitching motility, respectively. The latter two pathways were found to perform mechano- and surface sensing rather than chemosensing (28 – 30). Of the 18 chemoreceptors with a periplasmic LBD that belong to the F6 pathway (27), 10 have been functionality annotated (Table 1). Ligands of the other eight chemoreceptors involved in chemotactic behaviors are yet to be characterized, and given the variety of ligands that are typically sensed by a single LBD, even the annotated LBDs are likely to possess additional ligand specificities.

**TABLE 1:** The summary of 18 constructed hybrid chemoreceptors, their chemoeffectors, and pH responses

Locus tag	Name^*a*^	LBD type	Hybrid type^*b*^	Established chemoeffectors^*c*^	Newly characterized chemoeffectors^*d*^	pH response^*e*^	
						pH 6	pH 8
PA1251		4HB	T1, T3			—	—
PA1608	PctP	4HB	T1, T2		Guanine, inosine, adenosine, hypoxanthine, adenine, and guanosine	R	A
PA1646		HBM	T1, T3			—	—
PA2561	CtpH (31, 32)	4HB	T1, T3	Inorganic phosphate			
PA2573		4HB	T1			—	—
PA2652	CtpM (14)	sCache	T1, T2	Malate, +		—	—
PA2654	TIpQ (9)	dCache	T1	Histamine, +	2-Phenylethylamine, tyramine	R	R
PA2788	McpN (33)	PilJ	T1	Nitrate		—	—
PA2867		Unknown	T1, T3			—	—
PA2920		4HB	T1, T3			—	—
PA4307	PctC (34, 35)	dCache	T1, T2 (34)	γ-Aminobutyric acid (GABA), +	5-Aminovalerate, methyl 4-aminobutyrate	A	R
PA4309	PctA (35, 36)	dCache	T1, T2 (36)	L-alanine (L-Ala), +	L-ornithine (L-Orn) (37)^*f*^	A	R
PA4310	PctB (35, 36)	dCache	T1, T2 (36)	L-glutamine (L-Gln), +	L-ornithine (L-Orn) (37)^*f*^	—	—
PA4520		NIT	T1, T3				
PA4633	PctD (10)	dCache	T1, T2 (10)	Acetylcholine, +		—	—
PA4844	CtpL (31, 32)	HBM	T1	Inorganic phosphate		—	—
PA4915		4HB	T1		2-Phenylethylamine	—	—
PA5072	McpK (13)	HBM	T1, T3	α-Ketoglutarate, +		—	—

^*a*^ Ligand specificities of respective chemoreceptors have been characterized in indicated publications.

^*b*^ T1, type 1 is a hybrid chemoreceptor with a fusion site within TM2; T2: type 2 has a fusion site at the end of the HAMP domain; T3, type 3 has the same fusion site as type 1 but contains in addition a random linker of five amino acids. Hybrids that responds to D-glucose are underlined, and the nonresponsive hybrids are not underlined. For functional hybrid chemoreceptors that have been described previously, the references are provided in brackets.

^*c*^ Previously established ligands, with “+” indicating that the hybrid receptor was able to mediate a FRET response to this ligand.

^*d*^ Newly characterized chemoeffectors that showed positive results in FRET and/or ITC assays.

^*e*^ pH responses of hybrids, measured by changing buffer pH from 7 to either 6 or 8 using FRET assay. “A,” attractant response; “R,” repellent response; “—,” no response.

^*f*^ This study showed that PctA and PctB are required for chemotaxis of *P. aeruginosa* to L-ornithine, but no further characterization of L-ornithine binding to PctA or PctB was performed.

Several experimental approaches have been developed to identify ligands for bacterial chemoreceptors. The quantitative capillary chemotaxis assay is a traditional method for identifying bacterial chemoeffectors. However, because of differences in motility and physiology, the experimental conditions for the capillary assay need to be established for each individual bacterial strain, which hinders its general application. Moreover, the assignment of identified ligands to specific chemoreceptors typically requires the construction of strains with deletions of individual receptor genes and it is complicated by the frequent functional redundancy of multiple chemoreceptors. Alternatively, biochemical assays can be used for ligand identification *in vitro* (38). The thermal shift assay (TSA; alternatively called differential scanning fluorimetry [DSF]) can be applied to characterize the binding of chemical compounds to LBDs in high-throughput screens (39), but it is prone to yield false-positive results. Isothermal titration calorimetry (ITC) is, in contrast, an accurate but low-throughput method to measure ligand binding (40). Although a combination of these *in vitro* methods has proven to be very powerful for ligand identification (41), their application is limited to the structured LBD that can be purified and to compounds with high-affinity binding.

A complementary strategy for ligand identification relies on the construction of chimeric receptors that combine a periplasmic LBD of interest with the well-characterized output domain, such as that of the *E. coli* Tar receptor. Both chemoreceptor-chemoreceptor hybrids (42) and chemoreceptor-histidine kinase hybrids (43) that enable an *in vivo* readout of signaling response have been recently used to annotate unknown sensory functions. Here, we constructed a library of most *P. aeruginosa* periplasmic LBDs fused to the signaling domain of the *E. coli* chemoreceptor Tar and we used this library in combination with *in vivo* and *in vitro* assays to identify several novel physiologically relevant chemoeffectors and assign them to specific *P. aeruginosa* chemoreceptors. Overall, our screening strategy allowed us to expand the list of known chemoreceptor specificities for *P. aeruginosa* and a similar approach should be applicable to chemoreceptors from other bacteria and even to other types of receptors with a periplasmic LBD.

## RESULTS

### High-throughput screening for putative chemoeffectors in *P. aeruginosa* PAO1

To identify potential chemoeffectors for *P. aeruginosa*, we first screened chemical compounds from a large library of metabolites. Since several studies have shown correlation between a metabolic value of a compound and its potency as a chemoeffector (8, 44), we first used a growth assay to test the effects of chemical compounds from three plates of the commercial Biolog compound arrays (PM1, PM2A, and PM3B). This growth assay indeed showed that 202 compounds could be utilized as either carbon or nitrogen sources to support *P. aeruginosa* growth (Table S1). Out of those, we selected 29 compounds from Biolog compound arrays and we also included 10 compounds that have been either characterized as the specific ligands in other strains of *Pseudomonas* spp., share a structural similarity with established chemoeffectors, or are the ligands of other sensory systems in *P. aeruginosa* (13, 45 – 51). These 39 compounds have not yet been characterized as chemoattractants for *P. aeruginosa*, and we hypothesized that at least some of them are likely to be novel ligands for *P. aeruginosa* chemoreceptors.

**Fig 1:**
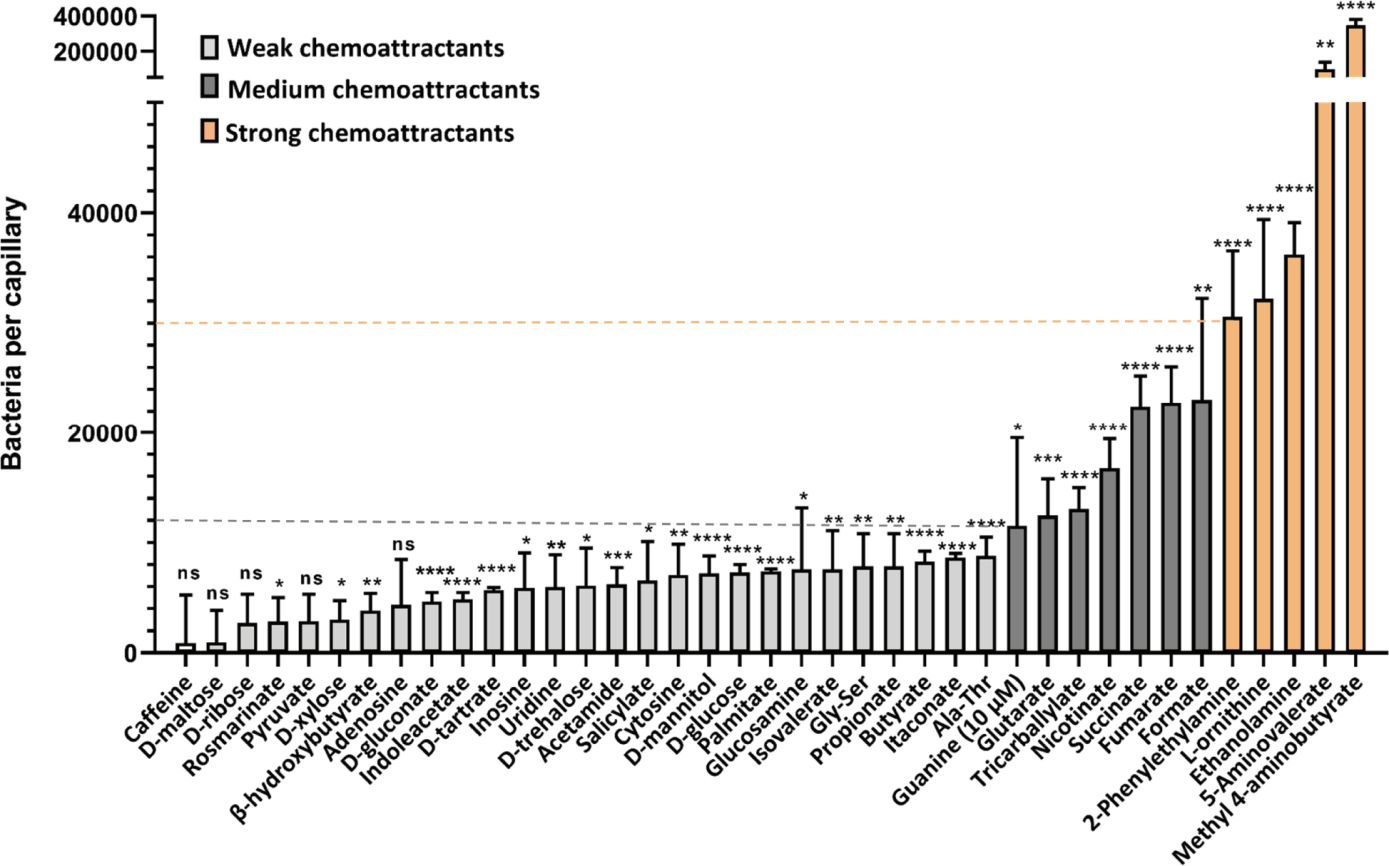
Chemotaxis of *P. aeruginosa* PAO1 toward potential chemoeffectors. Accumulation of bacteria (WT-Washington) in capillaries containing 1 mM of the indicated chemical compound in the chemotaxis buffer, except guanine that was used at 10 µM due to poor solubility. As indicated by dashed lines and colors, we defined compounds that attract ≥30,000 cells per capillary as strong chemoattractants (shown in orange); 10,000–30,000 cells per capillary as medium chemoattractants (shown in dark-gray) and <10,000 cells per capillary as weak chemoattractants (shown in light-gray). All data have been corrected by the number (7,825 ± 623) of bacteria in buffer-containing capillaries. The means and standard deviations of four biological replicates each conducted in triplicate are shown. Error bars indicate the mean ± standard deviations. Significance of difference from buffer-containing capillaries, assessed using an unpaired Student’s *t*-test, is indicated by asterisks (ns: nonsignificant, *P ≤ 0.05, **P ≤ 0.01, ***P ≤ 0.001, and ****P ≤ 0.0001).

We investigated the chemotactic responses of *P. aeruginosa* to these potential ligands by performing quantitative capillary chemotaxis assays (Fig. 1). For the initial high-throughput ligand screening, we tested all compounds at a fixed concentration of 1 mM as described previously (39, 52), besides guanine that was used at 10 µM due to a reduced solubility. At this concentration, five compounds were highly efficient in attracting bacteria, with the strongest responses being observed for methyl 4-aminobutyrate, followed by 5-aminovalerate, ethanolamine (EA), L-ornithine, and 2-phenylethylamine (PEA). Seven other compounds, guanine, glutarate, tricarballylate, nicotinate, succinate, fumarate, and formate, acted as chemoattractants of intermediate strength. The remaining compounds mediated only weak or no chemotaxis, despite being nutrients.

### Construction of chimeras for 18 transmembrane chemoreceptors in *P. aeruginosa* PAO1

**Fig 2:**
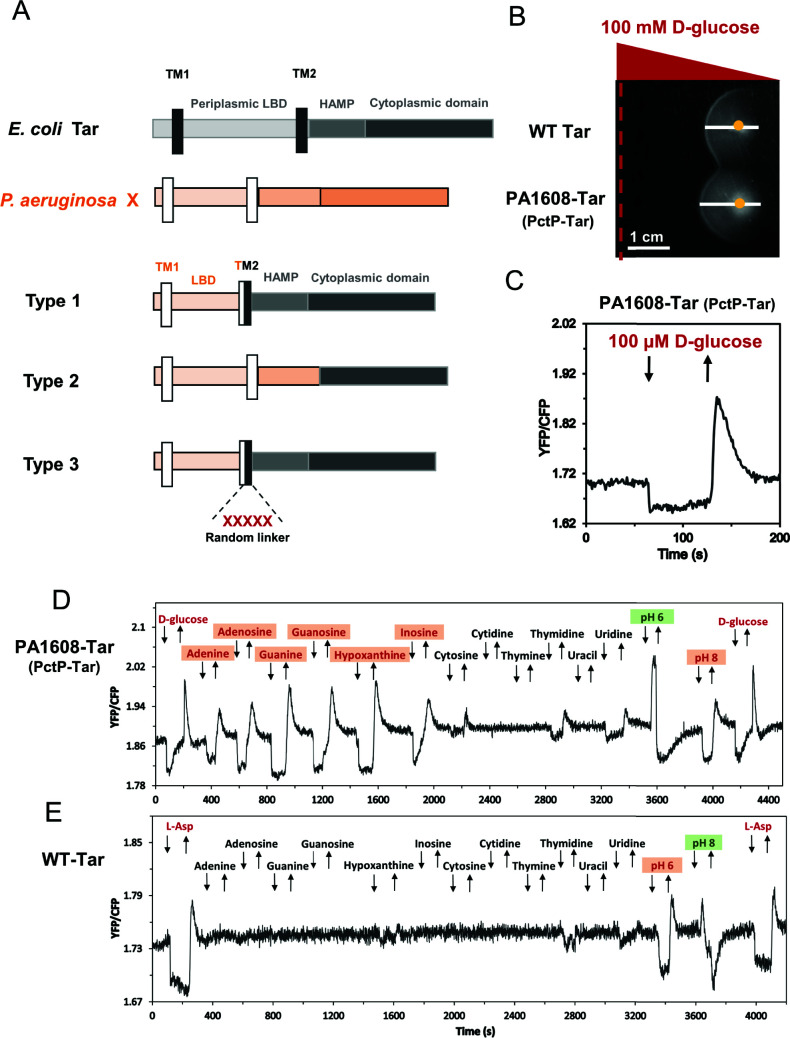
FRET-based screening of unassigned potential ligands using hybrid chemoreceptors. (**A**) Construction of three different types of chimeras by fusing the periplasmic domain of *P. aeruginosa* PAO1 chemoreceptors (X) and the cytoplasmic domain of *E. coli* Tar receptor. Each (hybrid) receptor consists of the periplasmic ligand binding domain, HAMP domain, and cytoplasmic domain, shown in different colors. The two transmembrane (TM) helices are shown by black and white rectangles for Tar and the target chemoreceptor (X), respectively. (**B**) D-glucose gradient plate assay was used for the assessment of receptor responsiveness to stimuli. Hybrid receptors were expressed as the sole receptor in *E. coli* cells, with the strain expressing wild-type Tar used as a positive control. Positions of the gradient source and of the cell inoculation site are shown by a red dashed line and by a yellow dot, respectively. Spreading of cells toward or away from the ligand source is indicated by the white line (scale bar, 1 cm). (**C**) FRET measurement of a hybrid chemoreceptor response to D-glucose. *E. coli* cells expressing the CheZ-CFP/CheY-YFP FRET pair and PA1608-Tar as the sole receptor are pre-adapted in tethering buffer responded to stepwise addition (down arrow) and subsequent removal (up arrow) of 100 µM D-glucose. Similar to canonical chemoattractants, addition of D-glucose inhibits pathway activity and thus lowers the YFP/CFP fluorescence ratio. (**D, E**) Examples of FRET measurements of *E. coli* FRET strain expressing PA1608-Tar (**D**) or wild-type Tar (**E**) as the sole receptor, responding to the stepwise addition (down arrow) and subsequent removal (up arrow) of indicated chemical compounds at a concentration of 100 µM or to pH change between default pH 7.0 and indicated pH. The activity of the PA1608-Tar hybrid was confirmed using D-glucose before screening potential ligands. Similarly, L-aspartate (L-Asp) was used as a positive ligand for wild-type Tar. The chemoattractants and chemorepellents are indicated in red and in green, respectively, with newly identified chemoeffectors being shaded.

To investigate the specificities of these unassigned chemoeffectors, we focused on the 18 transmembrane chemoreceptors that belong to the F6-type chemotaxis pathway of *P. aeruginosa* (Table 1). To increase the probability of obtaining a functional hybrid chemoreceptor for each candidate, we applied three previously described construction strategies that differ in the design of fusion sites (Fig. 2A): within the transmembrane (TM) helix 2 (type 1), after the HAMP domain (type 2), and within the TM2 helix with a five-amino acid random linker (type 3) (17, 42). The resulting hybrids of type 1 and type 3 connect the periplasmic LBD, the TM1 helix, and part of the TM2 helix from the *P. aeruginosa* chemoreceptor to the rest of the TM2 helix, the HAMP domain, and the cytoplasmic domain of *E. coli* chemoreceptor Tar. The fusion site of type 2 chimera is located in the junction between the HAMP domain and cytoplasmic domain, thus connecting the periplasmic LBD, the two TM helices, and the whole HAMP domain from the *P. aeruginosa* chemoreceptor to the cytoplasmic domain of Tar.

We subsequently tested the functionality of these chimeras expressed in a receptorless *E. coli* strain. We first used soft-agar gradient plates, where chemotactic cells exhibit biased spreading in gradients of compounds that are established by diffusion (Fig. 2B). Subsequently, we performed Förster resonance energy transfer FRET measurements (Fig. 2C) that are based on the phosphorylation-dependent interaction between CheY fused to yellow fluorescent protein (CheY-YFP) and CheZ fused to cyan fluorescent protein (CheZ-CFP). This assay enables to monitor activity of the chemotaxis pathway by following changes in the ratio of YFP/CFP fluorescence, which is proportional to CheA activity (53). Because the ligand specificity of many tested chemoreceptors is not known, D-glucose was routinely used as a nonspecific chemoattractant to assess the activity of hybrids and their responsiveness to stimuli. Differently from conventional chemoattractants, D-glucose is sensed via the phosphotransferase system (PTS) which signals to the cytoplasmic part of the chemoreceptor (54 – 56). A response to D-glucose thus demonstrates that the hybrid receptor activates the pathway and that it can be controlled by stimulation, but the functionality of periplasmic LBD and signal transduction toward the cytoplasmic part of the receptor remains to be confirmed by a specific ligand. From the constructed hybrids, only PA2561-Tar and PA4520-Tar showed no response to D-glucose. The functionality of several hybrids with already characterized periplasmic LBDs was further verified by measuring FRET responses to their established ligands (Table 1). Collectively, 16 active and glucose-responsive hybrid chemoreceptors were constructed successfully, and for seven of them, the functionality was further confirmed by stimulation with their established ligands.

### Screening of specific ligands for chemoreceptor chimeras using FRET

For these 16 active hybrids, we conducted the one-by-one screening with potential ligands at a fixed concentration of 100 µM, using FRET measurements in *E. coli* strains expressing the indicated hybrid chemoreceptor as a sole chemoreceptor along with the CheZ-CFP/CheY-YFP FRET pair. A number of tested ligands elicited similar responses for all (or most) receptor hybrids, as well as for the full-length Tar, suggesting that these stimuli might be sensed by the cytoplasmic domain of Tar (2) (Table S2). Responses to other potential ligands were hybrid specific, indicating their selectivity for a particular LBD (Table 1; Table S2). An example measurement for a hybrid that contains a previously uncharacterized 4HB-type LBD of PA1608 (Fig. 2D) showed responses to adenine, guanine, and inosine. These compounds did not stimulate Tar (Fig. 2E; Table S2), suggesting that the periplasmic LBD of PA1608 is a specific sensor of purines. Indeed, the PA1608-Tar hybrid showed a specific response to the other three purine derivatives but no or only minor response to pyrimidines, which were tested in this case in addition to our standard list of potential ligands (Fig. 2D and E). PA1608 was thus subsequently renamed as PctP (*Pseudomonas* chemotaxis transducer for purines; see below). Other positive ligands included L-ornithine (specific to PctA and PctB), methyl 4-aminobutyrate, and 5-aminovalerate (specific to PctC), which show structural similarity to the established ligands of the respective receptors (Table 1).

### Responses to external pH are mediated by the periplasmic domains of multiple chemoreceptors

**Fig 3:**
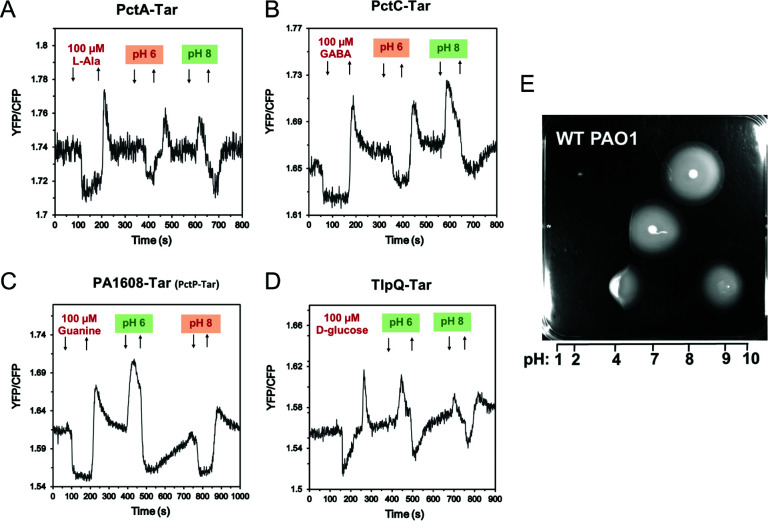
Responses to pH mediated by periplasmic domains of multiple *P. aeruginosa* chemoreceptors. (**A–D**) FRET response measurements for *E. coli* cells that expressed PctA-Tar (**A**), PctC-Tar (**B**), PctP-Tar (**C**), or TlpQ-Tar (**D**) as the sole chemoreceptor to the indicated changes in pH from default 7.0, which were performed as in Fig. 2. L-Ala (L-alanine), GABA (γ-aminobutyrate), guanine, and D-glucose were used as positive controls for PctA-Tar, PctC-Tar, PctP-Tar, and TlpQ-Tar, respectively. (**E**) pH taxis of *P. aeruginosa* PAO1 colonies that were inoculated at different points of the soft-agar plate with the established pH gradient. Estimated pH values along the gradient are indicated.

In addition to molecular compounds, we tested the response of hybrid chemoreceptors to external pH that is also an important stimulus for bacterial chemotaxis (18 – 20). In *E. coli*, a bidirectional taxis towards an intermediate pH is ensured by the push-pull action of an acid-seeking response mediated by Tar and a base-seeking response mediated by Tsr (20). Although Tar is able to sense both external (periplasmic) and internal (cytoplasmic) pH, moderate changes in external pH are sensed by the periplasmic domain (20, 57). We observed that, when initially adapted at neutral pH 7, PctA-Tar and PctC-Tar showed an attractant response to pH 6 and a repellent response to pH 8 (Fig. 3A and B), suggesting that these receptors mediate chemoattraction to acidic pH (i.e., acid-seeking behavior), whereas PctP-Tar showed a base-seeking repellent response to pH 6 and an attractant response to pH 8 (Fig. 2D and Fig. 3C). Interestingly, TlpQ-Tar showed repellent responses to both high and low pH (Fig. 3D), although the response to pH 8 was weaker. Such opposing pH responses mediated by different LBDs indicate that *P. aeruginosa* might exhibit bidirectional navigation in pH gradients in order to accumulate toward optimal pH, similar to the behavior of *E. coli* (20) and of *B. subtilis* (19). An accumulation of *P. aeruginosa* to an intermediate pH could be indeed observed when following spreading on soft-agar plates with a pH gradient (Fig. 3E).

### Characterization of PctP (PA1608) as a purine-specific chemoreceptor

**Fig 4:**
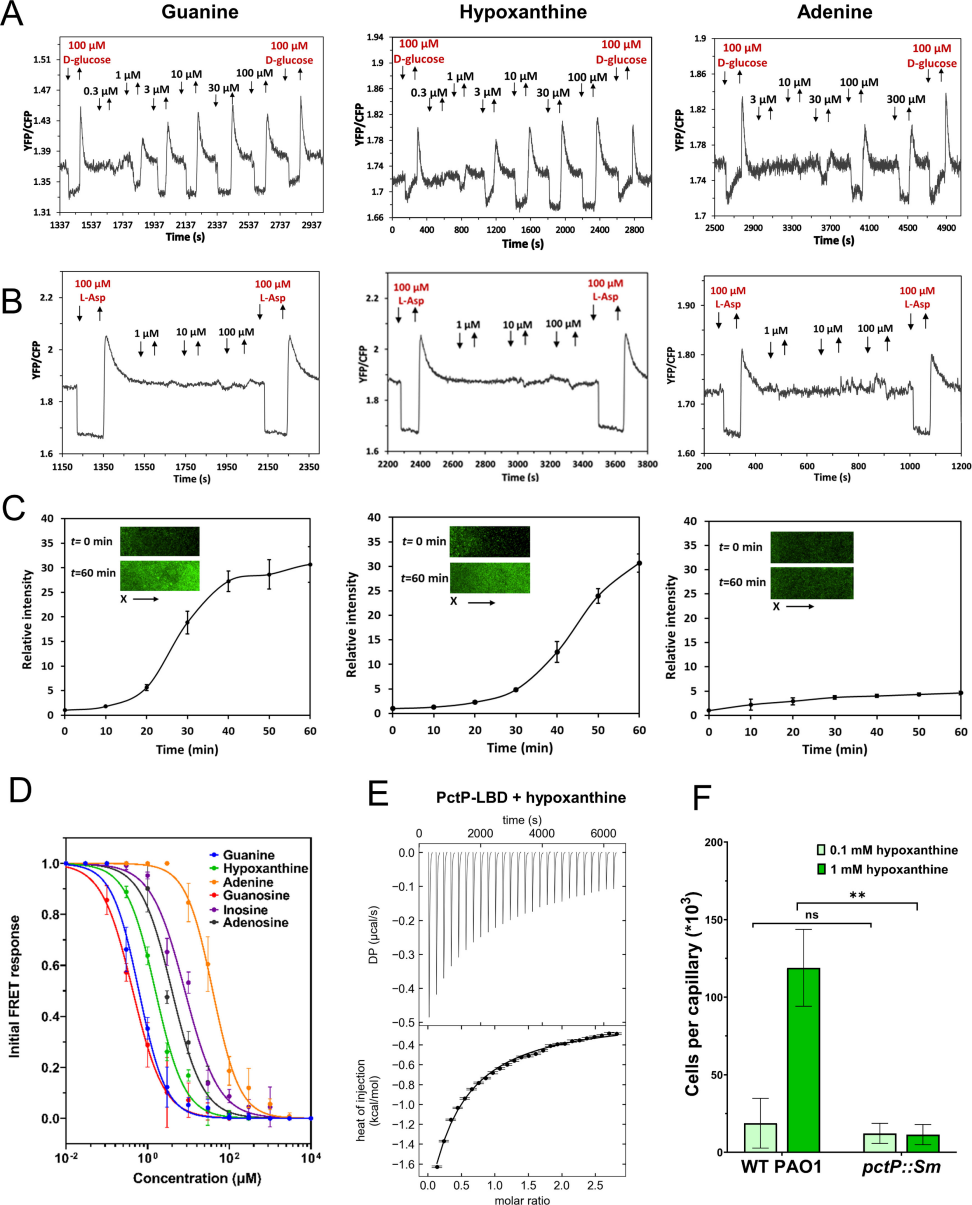
Characterization of PA1608 (PctP) as a purine-specific chemoreceptor. (**A, B**) FRET measurements for *E. coli* cells expressing PctP-Tar hybrid (**A**) or Tar receptor (**B**) as a sole receptor to the indicated concentrations of guanine, hypoxanthine, or adenine. FRET measurements were performed as described in Fig. 2. (**C**) Microfluidic assay of the chemotactic response of *E. coli* expressing PctP-Tar as the sole receptor and GFP as a label. Relative cell density (fluorescence intensity of GFP) in the observation channel over time in gradients of guanine, hypoxanthine, or adenine (in the same order as in A and B), with 50 mM in the source channel as indicated. Cell density in the observation channel before ligand stimulation (*t* = 0) was used to normalize all data. Error bars indicate standard deviation of three independent biological replicates. Insets show representative images of the observation channel at the beginning and the end of an experiment. The x-components (black arrow) indicate the direction up the concentration gradient. (**D**) Dose-response curves for FRET measurements of responses mediated by PctP-Tar. The amplitudes of the initial FRET response were calculated from changes in the ratio of YFP/CFP fluorescence after stimulation with indicated ligand concentrations and normalized to the saturated response. Error bars indicate the standard errors of three independent experiments; wherever invisible, error bars are smaller than the symbol size. Data were fitted using the Hill equation, and the EC_50_ fit values are shown in Table 2. (**E**) Microcalorimetric titrations of PctP-LBD with hypoxanthine. The upper panel shows the raw titration data, and the lower panel shows the integrated dilution-heat corrected and concentration-normalized peak areas fitted with the model for binding with negative cooperativity to two symmetric sites. Further experimental details are shown in Table S4. (**F**) Capillary chemotaxis assays of *P. aeruginosa* PAO1 and a *pctP* mutant to 0.1 mM and 1 mM hypoxanthine. The *pctP* mutant was derived from the Washington parental strain that was used as a WT PAO1 in this measurement. Data are shown as the means and standard deviations three biological replicates each conducted in triplicate. Significance of difference, assessed using a paired *t*-test, is indicated by asterisks (ns: nonsignificant, **P ≤ 0.01).

Our initial FRET screening showed that the PA1608-Tar hybrid responded to purine nucleobases and nucleosides but not to pyrimidines (Fig. 2D; Table S2), suggesting that PA1608 is a purine-specific chemoreceptor that was therefore renamed PctP. Binding of purines to the periplasmic LBD of PctP was further confirmed by TSA measurements (Fig. S1). We therefore investigated the chemotactic responses of the PctP-Tar hybrid to purine derivatives in greater detail. This hybrid receptor mediated responses to guanine and hypoxanthine in the lower micromolar range and to adenine at higher micromolar concentrations (Fig. 4A; Table 2). Consistently, chemotactic responses were seen for *E. coli* strain expressing PctP-Tar in gradients of guanine and hypoxanthine, but not of adenine, in the microfluidic chemotaxis assay (Fig. 4C). FRET responses were also observed for PctP-Tar expressing cells to all three corresponding purine nucleosides (Fig. S2A). No responses to these purine derivatives were observed for the *E. coli* expressing only the wild-type Tar receptor, as measured by FRET (Fig. 4B; Fig. S2B) or microfluidics (Fig. S3). Overall, the periplasmic LBD of PctP is more sensitive to guanosine, guanine, and hypoxanthine, followed by adenosine and inosine and finally by adenine (Fig. 4D; Table 2).

**TABLE 2:** Effective stimulatory concentration and dissociation constants for newly characterized chemoeffectors

Chemoreceptor	Chemoeffector	EC_50_ by FRET^*a*^ (µM)	*K_D_* by ITC^*b*^ (µM)
PA1608 (PctP)	Guanine	0.6 ± 0.1	
	Guanosine	0.4 ± 0.05	
	Hypoxanthine	1.6 ± 0.2	*K_D1_* = 43, *K_D2_* = 286
	Inosine	8.2 ± 1.9	
	Adenine	39.5 ± 4.5	
	Adenosine	3.9 ± 0.8	
PA2654 (TlpQ)	2-Phenylethylamine		410 ± 12
	Tyramine		100 ± 7
PA4307 (PctC)	5-Aminovalerate	1.5 ± 0.2	28 ± 2
	Methyl 4-aminobutyrate	2.9 ± 0.4	129 ± 2
PA4309 (PctA)	L-Ornithine (L-Orn)	1.8 ± 0.2	7.1 ± 0.5
PA4310 (PctB)	L-Ornithine (L-Orn)	10.2 ± 0.8	559 ± 80
PA4915	2-Phenylethylamine		58 ± 11

^*a*^ Half-maximal inhibitory ligand concentration (EC_50_) derived from dose-response curve measured by FRET.

^*b*^ Dissociation constants (*K_D_*) obtained from ITC measurements.

To test the potential physiological relevance of the observed preference of PctP for guanine compared with adenine, we grew *P. aeruginosa* in LB medium supplemented with either purine nucleobases or nucleosides. The addition of guanine or guanosine had growth-promoting effect, while adenine and adenosine rather had a slight inhibitory effect, apparently consistent with the ligand preference in the chemotaxis assay (Fig. S4).

Additional evidence for the binding of purines to PctP was obtained by ITC. It showed that the LBD of PctP binds hypoxanthine, with negative cooperativity (Fig. 4E; Table 2), while the binding of guanine and other purine derivatives was not detected due to the limited solubility or low binding affinity. Since hypoxanthine was not included in our initial high-throughput screening for identifying putative chemoeffectors, we have generated the *pctP* mutant and performed capillary assays of chemotaxis to hypoxanthine using the wild-type PAO1 and the mutant strain. Indeed, the wild-type PAO1 showed a strong chemoattractant response to 1 mM hypoxanthine, whereas the inactivation of the PctP chemoreceptor nearly abolished this response (Fig. 4F).

### Characterization of additional chemoeffectors for three amino acid chemoreceptors PctA, PctB and PctC

**Fig 5:**
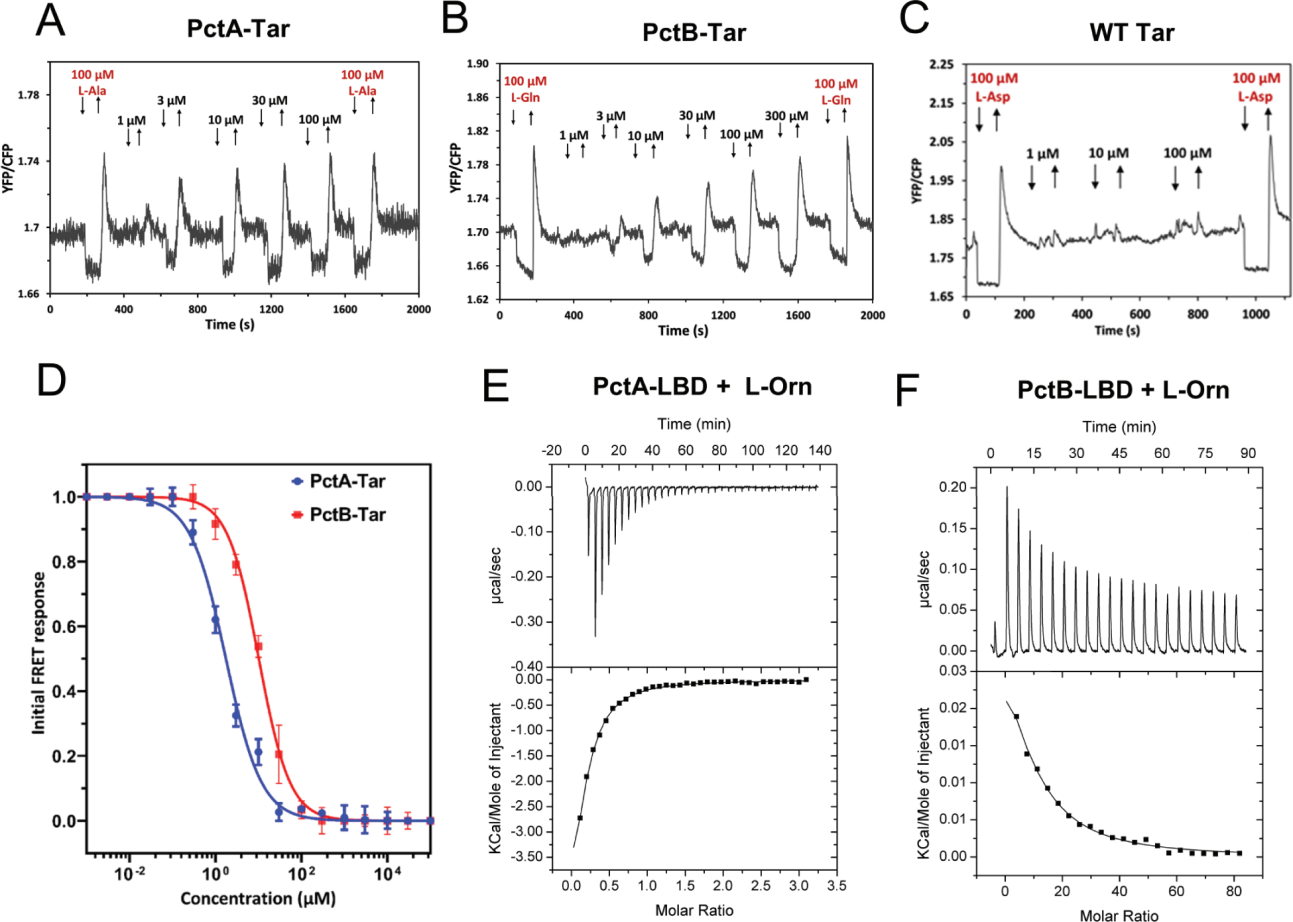
Characterization of L-ornithine sensing by PctA and PctB. (**A–D**) FRET response measurements for *E. coli* expressing PctA-Tar (**A**), PctB-Tar (**B**), or Tar receptor (**C**) as a sole receptor to the indicated concentrations of L-ornithine (L-Orn), and corresponding dose-response curves fitted using Hill equation (**D**). L-Ala (L-alanine), L-Gln (L-glutamine), and L-Asp were used as positive controls for PctA-Tar, PctB-Tar, and Tar, respectively. Error bars indicate the standard errors of three independent experiments; wherever invisible, error bars are smaller than the symbol size. The EC_50_ fit values are shown in Table 2. (**E, F**) Microcalorimetric titrations of PctA-LBD (**E**) and PctB-LBD (**F**) with L-Orn. The upper panels show the raw titration data, and lower panels show the integrated and corrected peak areas of the titration data that were fitted with the single-site binding model. Further experimental details are provided in Table S4.

The FRET results further showed that, besides their known amino acid ligands, PctA-Tar and PctB-Tar were responsive to L-ornithine (Fig. 5A and B), whereas no response could be observed for Tar (Fig. 5C). This observation is consistent with a recent report (37), although direct binding of L-ornithine to these receptors was not demonstrated in that previous study. Moreover, we observed that sensitivity to L-ornithine was higher for the response mediated by PctA-Tar. (Fig. 5D). We therefore performed ITC experiments using the purified LBDs of PctA and PctB, which confirmed binding of L-ornithine to both LBDs, with higher affinity for PctA than for PctB (Fig. 5E and F). This difference in affinity was consistent with the relative potency of these ligands as chemoeffectors for hybrids in *E. coli* (Table 2), when taking into account the *in vivo* signal amplification by the *E. coli* chemotaxis system.

**Fig 6:**
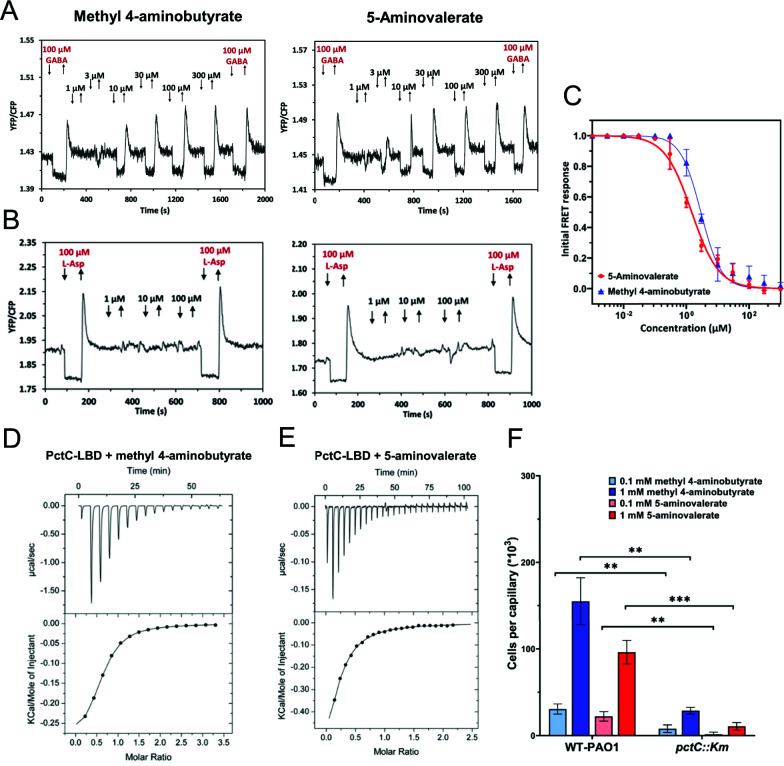
Characterization of PctC as a chemoreceptor for methyl 4-aminobutyrate and 5-aminovalerate. (**A–C**) FRET response measurements for *E. coli* cells expressing PctC-Tar hybrid (**A**) and Tar receptor (**B**) as a sole receptor to the indicated concentrations of methyl 4-aminobutyrate or 5-aminovalerate and corresponding dose-response curves of the PctC-Tar hybrid (**C**). Data were fitted using the Hill equation, and the EC_50_ fit values are summarized in Table 2. Error bars indicate the standard errors of three independent experiments; wherever invisible, error bars are smaller than the symbol size. (**D, E**) Microcalorimetric titrations of PctC-LBD with methyl 4-aminobutyrate (**D**) and 5-aminovalerate (**E**). The upper panels show raw titration data, and the lower panels show integrated and corrected peak areas of the titration data that were fitted with the single-site binding model. Further experimental details are provided in Table S4. (**F**) Capillary chemotaxis assays of the wild-type *P. aeruginosa* (WT-Hiroshima) and a *pctC* mutant strain (derived from WT-Hiroshima) response to methyl 4-aminobutyrate or 5-aminovalerate. Data are shown as the means and standard deviations of three biological replicates each conducted in triplicate. Significance of difference, assessed using a paired *t*-test, is indicated by asterisks (**P ≤ 0.01, ***P ≤ 0.001).

Similarly, the specificities of the PctC-Tar response to methyl 4-aminobutyrate and 5-aminovalerate were confirmed by the dose-response FRET measurements (Fig. 6A through C). We further demonstrated direct binding of methyl 4-aminobutyrate (Fig. 6D) and 5-aminovalerate (Fig. 6E) to the LBD of PctC by ITC, with dissociation constants of 129 and 28 µM, respectively. Again, these values were consistent with the higher sensitivity of PctC-Tar to 5-aminovalerate in FRET measurements (Table 2), taking in consideration the higher sensitivity of the *in vivo* response as discussed above. Finally, the physiological relevance of the PctC-mediated response for chemotaxis of *P. aeruginosa* towards methyl 4-aminobutyrate and 5-aminovalerate was confirmed by the capillary chemotaxis assays, where the *pctC* mutant strain showed strongly reduced chemotaxis compared with the wild-type strain (Fig. 6F).

### Characterization of chemoreceptors for ethanolamine, 2-phenylethylamine, and other biogenic amines

**Fig 7:**
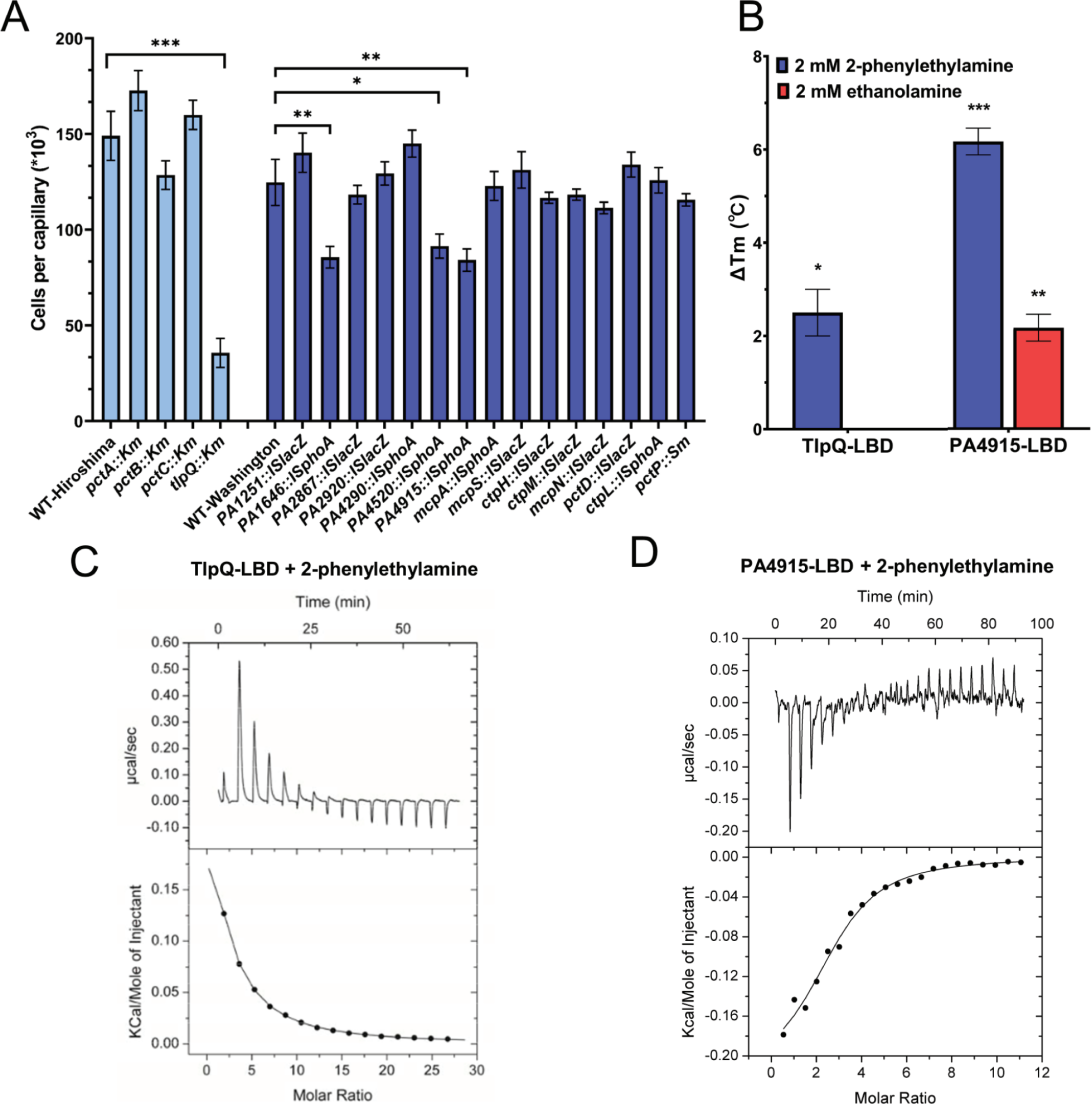
Identification of the chemoreceptors for ethanolamine and 2-phenylethylamine. (**A**) Capillary assay for chemotaxis toward 20 mM 2-phenylethylamine (PEA) for different chemoreceptor mutants of *P. aeruginosa* PAO1. Mutant strains deficient in PctA, PctB, PctC, and TlpQ were derived from WT-Hiroshima parental strain (shown in light-blue), which was used as a reference. The remaining mutant strains were derived from WT-Washington parental strain (shown in blue), which was used as a reference for these mutants. Data are shown as the means and standard deviations of three biological replicates each conducted in triplicate. Significance of difference from buffer-containing capillaries, assessed using an unpaired Student’s *t*-test, is indicated by asterisks (*P ≤ 0.05, **P ≤ 0.01, and ***P ≤ 0.001). (**B**) Thermal shift assay for TlpQ-LBD and PA4915-LBD in the presence of 2 mM PEA or ethanolamine (EA). Data are shown as the means and standard deviations of three biological replicates each conducted in triplicate. Significance of difference from zero, assessed by one sample *t*-test, is indicated by asterisks (*P ≤ 0.05, **P ≤ 0.01, and ***P ≤ 0.001). (**C, D**) Microcalorimetric titrations of TlpQ-LBD (**C**) and PA4915-LBD (**D**) with PEA. Upper panels show raw titration data, whereas lower panels show the best fit of the integrated, concentration-normalized, and dilution-heat corrected raw data using the single-site binding model. Further experimental details are provided in Table S4.

Two remaining strong chemoattractants, ethanolamine (EA) and 2-phenylethylamine (PEA), did not elicit apparent responses in our initial FRET screening. In order to identify their specific chemoreceptor(s), we screened receptor mutant strains of *P. aeruginosa* in the presence of 20 mM EA and PEA using quantitative capillary assays. Although no conclusive results were obtained for EA (data not shown), a significant decrease in the chemoattraction to PEA was observed for the *tlpQ* mutant strain and intermediate reductions for PA1646, PA4520, and PA4915 mutant strains (Fig. 7A), indicating that these receptors might play a role in the chemotaxis to PEA. This was supported by the thermal shift assays, which suggested that TlpQ-LBD and PA4915-LBD might bind PEA and PA4915-LBD might also bind EA (Fig. 7B). In these cases, we were unable to observe clear FRET responses of TlpQ-Tar and PA4915-Tar hybrids to PEA or EA, although these two hybrids showed good responses to D-glucose (Table S2) and TlpQ-Tar mediated (weak) attractant responses to its known ligands such as histamine, spermidine, and spermine (Fig. S5A). This suggests that TlpQ-Tar and PA4915-Tar hybrids are not fully functional, explaining why responses to PEA and EA were not detected in our initial FRET screen. Nevertheless, our ITC measurements confirmed the direct binding of PEA to TlpQ-LBD and PA4915-LBD (Fig. 7C and D; Table 2). Collectively, despite poor functionality of their hybrids, we conclude that TlpQ and PA4915 are the major chemoreceptors for PEA chemotaxis in *P. aeruginosa*.

In previous studies, TlpQ was demonstrated to bind several biogenic amines, including histamine, spermine, agmatine, cadaverine, and putrescine (9). Since some of these compounds, as well as the newly characterized ligand—PEA, are produced by the decarboxylation of amino acids, we speculated that TlpQ might also sense tyramine, the decarboxylation product of tyrosine. Indeed, the *tlpQ* mutant strain showed much-reduced chemotaxis toward tyramine in the capillary assay (Fig. S5B) and binding of tyramine to TlpQ-LBD was further confirmed by ITC (Table 2; Fig. S5C).

Finally, we used FRET to test responses of three amino acid chemoreceptors PctA, PctB, and PctC to histamine, one of the most important biogenic amines (58, 59). A recent study (9) suggested that next to TlpQ, PctA and PctC also participate in the histamine chemotaxis but microcalorimetric titrations of the corresponding LBDs revealed only binding to TlpQ. In contrast, FRET measurements confirmed that PctB and PctC could sense histamine in the medium micromolar range and in the high micromolar range, respectively (Fig. S6), highlighting the advantage of using receptor hybrids for characterization of low-affinity ligands.

## DISCUSSION

Bacteria contain an extensive array of different sensory receptors that respond to a variety of stimuli, regulating multiple physiological functions including gene expression, chemotaxis, or second messenger signaling. Major receptor families include sensory histidine kinases, chemoreceptors, adenylate, diadenylate and diguanylate cyclases, and phosphodiesterases, as well as Ser/Thr/Tyr protein kinases and phosphoprotein phosphatases (60). Typically, these receptors are stimulated by the binding of signal molecules to their sensory domains that contain all the requisites for ligand binding. However, the lack of established signals that are recognized by receptors is currently a major bottleneck in our understanding of signal transduction processes in bacteria (61).

The fact that the same type of periplasmic LBD is frequently found in different signal transduction systems suggests their modular nature and indicates that these domains have been exchanged among different receptor families during evolution. This notion is exemplified by the dCache-type domain that is the predominant type of the bacterial periplasmic LBDs, present in all major bacterial receptor families (62). Previous success with construction of hybrid receptors (42, 43, 63, 64) confirmed this modularity and indicated that the exchange of periplasmic LBDs between different receptors can also be reproduced in the laboratory, and it is likely that such hybrid construction could be extended beyond chemoreceptor-chemoreceptor and chemoreceptor-sensor kinase hybrids to sensory domains from other types of receptors. Ligand screening for hybrid receptors, as done in this work, has thus the promise to become a universal approach to identify receptor ligands and thus to tackle a major bottleneck in microbiology.

Here, we combined the screening based on hybrid chemoreceptors with binding and capillary assays to systematically identify novel ligands for chemoreceptors in *P. aeruginosa*, an important pathogen with a complex lifestyle and the correspondingly broad chemosensory range. Since many known bacterial chemoattractants are metabolically valuable compounds (8, 44), we first performed high-throughput screenings for candidate chemoeffectors using the growth assay, followed by an evaluation of their potency as chemoattractants for *P. aeruginosa* in the capillary chemotaxis assays. The strongest chemoattractants were then prioritized for further investigation using a library of hybrid chemoreceptors, containing the periplasmic LBDs of *P. aeruginosa* chemoreceptors fused to the cytoplasmic signaling domain of *E. coli* Tar receptor, to identify the potential ligand-receptor pairs using FRET and microfluidic assays (42). This approach has several advantages, including highly sensitive and standardized chemotaxis assays already established in *E. coli*, which enable detection not only of high- but also of low-affinity ligands and testing not only binding of ligands but also their signaling properties. It further avoids the complication of functional redundancy between *P. aeruginosa* chemoreceptors with overlapping ligand specificities. Nevertheless, this hybrid-based *in vivo* screening also has some limitations, primarily because most but not all of the constructed hybrids are functional, and in those cases, it was complemented by *in vitro* ligand screening using TSA. Direct binding between the high-affinity ligands and the LBD of chemoreceptors could be further confirmed using ITC. Finally, capillary chemotaxis assays were used to demonstrate the physiological relevance of identified ligand-receptor interactions for chemotaxis of *P. aeruginosa*. This combination of assays enabled us to identify new ligands for several previously studied chemoreceptors and to characterize a new chemoreceptor in this well-studied model organism (Table 1).

Our results showed that the novel purine-specific chemoreceptor of *P. aeruginosa* PctP has the highest affinity for guanosine, guanine, and hypoxanthine, intermediate affinity for adenosine, and significantly lower affinity for adenine. Although physiological significance of the PctP-mediated chemotactic response of *P. aeruginosa* to guanine and other purine derivatives remains to be elucidated, *P. aeruginosa* infection might be limited by low availability of nucleobases in the human host (65), including the growth of *P. aeruginosa* in the lungs of cystic fibrosis (CF) patients (66). Cross-feeding of purine derivatives might be important in a polymicrobial community in CF lungs (67). In apparent agreement with the ligand preference of PctP for guanine compared with adenine, we observed that the growth of *P. aeruginosa* in rich medium was promoted by the addition of guanine or guanosine, but not (or even negatively) affected by the addition of adenine or adenosine. Despite their potential importance, there are only few existing reports of chemotaxis towards purine or pyrimidine derivatives (11, 12, 68, 69) and only a single bacterial chemoreceptor specific for metabolizable purines has been characterized so far, McpH from *P. putida* KT2440 (69). Interestingly, despite the phylogenetic proximity of *P. aeruginosa* and *P. putida*, McpH possesses a dCache-type LBD, whereas PctP has a four-helix bundle-type LBD, indicative of convergent evolution (49) and providing further support for the important role of purine chemotaxis.

Besides characterizing a novel purine-specific chemoreceptor PctP, we assigned additional ligands to the characterized chemoreceptors. One example of the former category is the observed response to L-ornithine mediated by two amino acid chemoreceptors PctA and PctB, which support a recent study that implicated these receptors in *P. aeruginosa* chemotaxis towards L-ornithine (37). L-ornithine is a biologically versatile nonproteinogenic derivative of L-arginine. Besides its effects on growth, L-ornithine is known to promote *P. aeruginosa* biofilm formation (70). L-ornithine might also accumulate in the human lung environment during conditions that are associated with *P. aeruginosa* infections due to the elevated production of arginase (71), indicating a possible role of L-ornithine chemotaxis in virulence and making its receptors potentially attractive target for therapeutic interventions. Indeed, the mutation of *pctA*, *pctB*, and *pctC* reduced the accumulation of *P. aeruginosa* towards wounded lung epithelial cells (72). We observed that PctA senses L-ornithine at much lower concentrations than PctB, which is consistent with the previously observed difference in the sensitivity of these two receptors to L-arginine and several other amino acids (36). Another example is the *P. aeruginosa* response to other amino acid derivatives, methyl 4-aminobutyrate and 5-aminovalerate. We showed that PctC, the high-affinity receptor for gamma-aminobutyric acid (GABA), could sense these compounds. This is consistent with the structural similarity of these compounds to GABA, although their affinities to PctC-LBD are 10- to 100-fold lower than that of GABA (34, 35).

The responses to ethanolamine, 2-phenylethylamine, and tyramine further expand the ligand range of characterized chemoreceptors in *P. aeruginosa*. TlpQ was previously reported to bind several biogenic amines (BAs), including putrescine, histamine, agmatine, and cadaverine (9), which are derived from the decarboxylation of L-amino acids. BAs have important physiological functions in eukaryotic and prokaryotic cells, and many bacteria are able to produce and/or degrade BAs (59, 73). Here, with PEA and tyramine, we identified two additional TlpQ ligands. Sequence comparison between the dCache domain of TlpQ and the amino acid-binding dCache domains of PctA, PctB, and PctC (74) shows conservation of amino acids that bind the amine group, which may explain the capacity of these receptors to bind either amino acids or their decarboxylated derivatives. Furthermore, we identified a second, previously uncharacterized, receptor PA4915 as a sensor of PEA and possibly also of EA. The binding affinity of PA4915-LBD for PEA is even higher than that of TlpQ-LBD, suggesting that it might be particularly important to mediate response to low concentrations of PEA. Notably, PA4915 possesses the 4HB-type LBD, which provides an interesting example of receptors within one bacterium that harbor different types of LBDs but respond to the same ligand.

In addition to characterizing novel receptor specificities for molecular compounds, we observed that multiple periplasmic domains of *P. aeruginosa* chemoreceptors could mediate specific responses to external pH. In two model neutrophilic bacteria where pH taxis has been studied, *E. coli* (20) and *Bacillus subtilis* (19), bacterial accumulation toward neutral pH is ensured by opposite pH responses mediated by different receptors. In *E. coli*, the acid-seeking response is primarily mediated by the periplasmic domain of Tar and the base-seeking response is mediated by the periplasmic domain of Tsr. Our results suggest that bidirectional pH taxis of *P. aeruginosa* is even more complex, with the periplasmic domains of PctA and PctC mediating the acid-seeking behavior and those of TlpQ and PctP mediating the base-seeking behavior.

Taken together, our systematic screening strategy enabled us to enlarge the spectrum of ligand specificities of periplasmic domains in *P. aeruginosa*, despite it being already a well-studied model for bacterial chemotaxis. A similar strategy could be applied for the systematic characterization of unknown sensory domains from different types of receptors in other species, including those that are unculturable.

## MATERIALS AND METHODS

### Bacterial strains, plasmids, and culture conditions

Bacterial strains and plasmids are listed in Table S3. For chemotaxis and FRET experiments, *E. coli* was grown in TB medium (1% tryptone and 0.5% NaCl) at 34°C. For molecular cloning and protein expression, *E. coli* was grown in LB medium at 37°C. *P. aeruginosa* was grown overnight in M9 minimal medium containing 15 mM D-glucose at 37°C. When necessary, antibiotics were used at the following final concentrations: kanamycin, 50 µg/mL (*E. coli* strains); ampicillin, 100 µg/mL (*E. coli* strains); chloramphenicol, 34 µg/mL (*E. coli* strains) and 100 µg/mL (*P. aeruginosa* strains); and tetracycline, 50 µg/mL (*P. aeruginosa* strains).

### PA1608 mutant generation

To generate the *pctP* mutant strain, a 1,280bp fragment of PA1608 was amplified by PCR using primers PA1608_MUT_F and PA1608_MUT_R (Table S3). The PCR product was cloned into the pGEM-T vector and transformed into *E. coli* DH5α. The resulting plasmid pGEMT-PA1608 was digested with ApaI and SpeI and the insert cloned into pKNG101 previously digested with the same enzyme. The resulting plasmid pKNG101-PA1608 was then transformed into *E. coli* CC118 λpir. pKNG101_PA1608 was then introduced into *P. aeruginosa* PAO1 by electroporation according to reference 75. The mutant was verified by PCR and sequencing.

### Growth assay

The analysis of the nutritional profile was carried out using the “Phenotype Microarrays” plates PM1, PM2A, and PM3B (for further information, refer to https://www.biolog.com). Each of these plates contains 95 chemical compounds and one control (H_2_O). To determine the growth of *P. aeruginosa* using these compounds as the sole carbon or nitrogen source, the lyophilized compounds present on the Biolog plates were resuspended in 90 µL of either M9 medium (for Biolog PM1 and PM2A plates) or M8 medium (M9 minimal medium without NH_4_Cl) containing 15 mM glucose (for Biolog PM3B plate). Subsequently, a *P. aeruginosa* overnight culture was washed twice with M8 salt medium, diluted to an OD_600_ of 0.2 in M9 or M8 salt medium, and then, the wells with each of the compounds were inoculated with 10 µL of these cultures. Finally, growth was monitored at 37°C with shaking, determining the OD_600_ every hour for 48 hr in a Bioscreen Microbiological Growth Analyser (Oy Growth Curves Ab Ltd., Helsinki, Finland).

### Chemotaxis capillary assays for *P. aeruginosa* strains

Overnight cultures in M9 minimal medium supplemented with 6 mg/L Fe-citrate, trace elements (76), and 15 mM glucose were used to inoculate fresh medium to an OD_660_ of 0.05. Cells were cultured at 37°C to an OD_660_ of 0.4. Subsequently, cells were washed twice by centrifugation (1,667 × *g* for 5 min) and resuspended in chemotaxis buffer (50 mM KH_2_PO_4_/K_2_HPO_4_, 20 mM EDTA, and 0.05% [vol/vol] glycerol, pH 7.0). Aliquots (230 µL) of the cell suspension at an OD_660_ of 0.1. were placed into the wells of 96-well microtiter plates. Then, 1-µL capillaries (Microcaps, Drummond Scientific) were heat sealed at one end and filled with buffer (control) or chemoeffector solution prepared in chemotaxis buffer. The capillaries were rinsed with sterile water and immersed into the bacterial suspensions at their open ends. After 30 min, capillaries were removed from the wells, rinsed with sterile water, and emptied into 1 mL of chemotaxis buffer. Serial dilutions were plated onto M9 minimal medium plates supplemented with 20 mM glucose, incubated at 37°C prior to colony counting. Data were corrected with the number of cells that swam into buffer containing capillaries.

### Construction of hybrid chemoreceptors

To construct the hybrid type 1 in a high-efficient way, the dropout plasmid pHC8 was generated by the Golden Gate Assembly Kit (New England BioLabs) and the basic genetic parts are from Marburg Collection (77). The dropout part served as the placeholder, which carried a full expression cassette for the fluorescent proteins sfGFP to enable visible distinction of correct colonies and outward facing BsaI recognition sites. The signaling domain of Tar (198–553) was connected after the dropout part. The periplasmic domains of *P. aeruginosa* chemoreceptors containing BsaI recognition sites at both ends were synthesized, which allowed for replacing the dropout part by Golden Gate reaction, then generating the hybrid chemoreceptor in one-step reaction. For the constructions of hybrid type 2 and type 3, the coding sequences of each hybrid were amplified using PCR reaction (oligonucleotide sequences are shown in Table S3). The amplified fragments containing overlapping sequences of vector pKG116 were ligated into the digested vector pKG116 using Gibson assembly reaction in NEBuilder HiFi DNA Assembly Master Mix (New England BioLabs). After cloning, the active or functional hybrid type 3 was selected from a library of chemoreceptor[1–X]-XXXXX-[203–553] as described previously (42) .

### FRET measurements

FRET measurements were performed as described previously (42, 53, 78). Cultures of the receptorless *E. coli* strain VS181 expressing chimeras of interest and CheY-YFP/CheZ-CFP FRET pair were prepared by inoculating 200 µL of the overnight culture into 10 mL TB medium supplemented with appropriate antibiotics and inducers (50 µM isopropyl-β-D-thiogalactoside (IPTG) and 1–2 µM sodium salicylate) and grown in a rotary shaker at 34°C and 275 rpm. Cells were harvested at OD_600_ of 0.5 by centrifugation and washed twice with tethering buffer (10 mM KH_2_PO_4_/K_2_HPO_4_, 0.1 mM EDTA, 1 µM methionine, and 10 mM sodium lactate, pH 7.0). For microscopy, the cells were attached to the poly-lysine-coated coverslips for 10 min and mounted into a flow chamber that was maintained under constant flow of 0.3 mL/min of tethering buffer using a syringe pump (Harvard Apparatus) that was also used to add or remove compounds of interest. The pH value of all tested compounds was adjusted to 7, except for compounds that are only soluble under acidic or basic conditions, where the background buffer at corresponding pH was tested as a negative control. FRET measurements were performed on an upright fluorescence microscope (Zeiss AxioImager.Z1) equipped with photon counters (Hamamatsu). The fluorescence signals were recorded and analyzed as described previously (17).

### Protein overexpression and purification

The LBDs of chemoreceptors PctA, PctB, PctC, PctD, PA4915, and TlpQ were purified as described previously (9, 35). For the remaining proteins, the LBDs were cloned into a pET28b(+) expression vector. *E. coli* BL21 (DE3) harboring the LBD expression plasmid was grown in 5-L Erlenmeyer flasks containing 1 L LB medium supplemented with kanamycin under continuous stirring (200 rpm) at 37°C. When OD_600_ reached 0.6, 0.1 mM isopropyl-β-D-thiogalactoside (IPTG) was added to induce protein expression. Growth was continued at 16°C for 12 hr, and cells were harvested by centrifugation at 10,000 × *g* for 30 min at 4°C. Proteins were purified by metal affinity chromatography using modified procedures for the His GraviTrap column (Cytiva Lifesciences, Marlborough, MA, USA). Briefly, cell pellets were resuspended in binding buffer (20 mM sodium phosphate, 500 mM NaCl, and 20 mM imidazole, pH 7.4) supplemented with 0.2 µg/mL lysozyme, 1 mM MgCl_2_, and 1 mM PMSF, stirred for 30 min at 4°C, and lysed by an ultrasonic homogenizer for 10 min at 80% amplitude (SONOPULS HD 4000; BANDELIN electronic GmbH & Co. KG, Berlin, Germany). After centrifugation at 20,000 × *g* for 30 min, the supernatant was loaded into His GraviTrap column pre-equilibrated with binding buffer and target proteins were eluted by elution buffer (20 mM sodium phosphate, 500 mM NaCl, and 500 mM imidazole, pH 7.4). The protein fractions were dialyzed with the dialysis buffer (10 mM sodium phosphate, pH 5.5) to remove imidazole and NaCl and concentrated using Amicon Ultra-15 centrifugal filters (Merck Millipore Ltd., Burlington, MA, USA).

### Soft-agar plate assay for *E. coli* strains

To establish gradients, 200-µL aliquots of 100 mM chemical solutions were applied to the center line of minimal A agar plates (0.25% [wt/vol] agar, 10 mM KH_2_PO_4_/K_2_HPO_4_, 8 mM [NH4]_2_SO_4_, 2 mM citrate, 1 mM MgSO_4_, 0.1 mg/mL of thiamine-HCl, 1 mM glycerol, and 40 µg/mL of a mixture of threonine, methionine, leucine, and histidine) supplemented with antibiotics and inducers and incubated overnight at 4°C for gradient formation. The receptorless *E. coli* cells expressing the chimera of interest as a sole receptor were grown overnight in 5 mL TB medium, harvested by centrifugation, washed by tethering buffer, resuspended in 200 µL tethering buffer, and applied to the plate at a 2.5 cm distance from the line where the chemical was inoculated. Plates were incubated at 30°C for 24–48 hr.

The pH taxis assay was performed on semi-solid agar plates (0.27% Bacto-agar in TB). After solidification, 100 µL 37% HCl and 40% NaOH were applied on the left and right sides of the plate, respectively, to establish the pH gradient, and 2 µL of overnight cultures was inoculated on the plates with different distances to the central line of the plate. To enhance the formation of the pH gradient, 100 µL 37% HCl and 40% NaOH were added again on the same position after 8 hr of incubation at 30°C. Images of the plates were taken after 10–14 hr, and the pH of the medium at different positions was estimated using the pH indicator paper.

### Microfluidic assays

The microfluidic assay was performed as previously described, using a chip with 24 parallel microchannels (79). The receptorless *E. coli* strain UU1250 expressing GFP and chimera of interest were grown at 34°C in TB supplemented with antibiotics and inducers until OD_600_ of 0.5. Cells were harvested by centrifugation and washed twice with tethering buffer. The compounds of interest were dissolved in tethering buffer at a concentration of 50 mM, and the pH was adjusted to 7.0. The chemical source microchannels were filled with 4% (wt/vol) low-gelling temperature agarose to create a semi-permeable barrier. *E. coli* cells were added in the reservoir well and allowed to spread for 30 min into the channels. Compounds were added to the source well and allowed to form a concentration gradient. Cell fluorescence was recorded with a Nikon Ti-E inverted microscope system (Nikon Instruments Europe BV, Amsterdam, Netherlands) using a 20× objective. Data were analyzed using ImageJ (Wayne Rasband, National Institutes of Health, USA).

### Thermal shift assays

Thermal shift assays were performed in 384 microtiter plates using a Bio-Rad CFX384 Touch Real-Time PCR instrument with the presence or absence of potential chemoattractants. Each 25-µL assay mixture contained 20.5 µL purified protein (30–100 μM) in phosphate buffer (10 mM sodium phosphate, pH 5.5), 2 µL SYPRO Orange (Invitrogen by Thermo Fisher Scientific, Waltham, MA, USA) at 5× concentration, and 2.5 µL of 20-mM potential chemoattractant. Samples were heated from 23°C to 95°C at a scan rate of 1°C/min. The protein unfolding curves were monitored by detecting changes in SYPRO Orange fluorescence. The resulting data permitted the calculation of the mid-point of the protein unfolding transition or melting temperatures (Tm) using the first derivative values from the raw fluorescence data, which were analyzed by the Bio-Rad CFX manager 3.1 software.

### Isothermal titration calorimetry

Experiments were conducted on a VP-microcalorimeter (Microcal, Amherst, MA, USA) at the temperatures indicated in Table S4. Proteins were dialyzed into the buffer specified in Table S4 and placed into the sample cell. Ligand solutions were made up in dialysis buffer at the concentrations indicated in Table S4 and titrated into the protein. The mean enthalpies measured from the injection of ligands into buffer were subtracted from raw titration data prior to data analysis with the MicroCal version of ORIGIN. Data were fitted with the single-site binding model. In cases where data analysis with this model did not result in a satisfactory fit, data were analyzed in SEDPHAT (80) using a model for the binding with negative cooperativity to a macromolecule containing two symmetric sites.

## Data Availability

All of the data are included in this article.

## SUPPLEMENTAL MATERIAL

Supplemental Material mbio.02099-23-s0001.pdf Fig. S1 to S6; Tables S1 to S4

ASM does not own the copyrights to Supplemental Material that may be linked to, or accessed through, an article. The authors have granted ASM a non-exclusive, world-wide license to publish the Supplemental Material files. Please contact the corresponding author directly for reuse.

## References

[1] Sourjik V , Wingreen NS . 2012. Responding to chemical gradients: bacterial chemotaxis. Curr Opin Cell Biol 24:262–268. doi:10.1016/j.ceb.2011.11.008 22169400 PMC3320702

[2] Bi S , Sourjik V . 2018. Stimulus sensing and signal processing in bacterial chemotaxis. Curr Opin Microbiol 45:22–29. doi:10.1016/j.mib.2018.02.002 29459288

[3] Keegstra JM , Carrara F , Stocker R . 2022. The ecological roles of bacterial chemotaxis. Nat Rev Microbiol 20:491–504. doi:10.1038/s41579-022-00709-w 35292761

[4] Colin R , Ni B , Laganenka L , Sourjik V . 2021. Multiple functions of flagellar motility and chemotaxis in bacterial physiology. FEMS Microbiol Rev 45:fuab038. doi:10.1093/femsre/fuab038 34227665 PMC8632791

[5] Hazelbauer GL , Falke JJ , Parkinson JS . 2008. Bacterial chemoreceptors: high-performance signaling in networked arrays. Trends Biochem Sci 33:9–19. doi:10.1016/j.tibs.2007.09.014 18165013 PMC2890293

[6] Ortega Á , Zhulin IB , Krell T . 2017. Sensory repertoire of bacterial chemoreceptors. Microbiol Mol Biol Rev 81:e00033-17. doi:10.1128/MMBR.00033-17 PMC5706747 29070658

[7] Mesibov R , Adler J . 1972. Chemotaxis toward amino acids in Escherichia coli. J Bacteriol 112:315–326. doi:10.1128/jb.112.1.315-326.1972 4562400 PMC251414

[8] Yang Y , M Pollard A , Höfler C , Poschet G , Wirtz M , Hell R , Sourjik V . 2015. Relation between chemotaxis and consumption of amino acids in bacteria. Mol Microbiol 96:1272–1282. doi:10.1111/mmi.13006 25807888 PMC5008178

[9] Corral-Lugo A , Matilla MA , Martín-Mora D , Silva Jiménez H , Mesa Torres N , Kato J , Hida A , Oku S , Conejero-Muriel M , Gavira JA , Krell T . 2018. High-affinity chemotaxis to histamine mediated by the TlpQ chemoreceptor of the human pathogen Pseudomonas aeruginosa. mBio 9:e01894-18. doi:10.1128/mBio.01894-18 30425146 PMC6234866

[10] Matilla MA , Velando F , Tajuelo A , Martín-Mora D , Xu W , Sourjik V , Gavira JA , Krell T . 2022. Chemotaxis of the human pathogen Pseudomonas aeruginosa to the neurotransmitter acetylcholine. mBio 13:e0345821. doi:10.1128/mbio.03458-21 35254130 PMC9040839

[11] Liu X , Parales RE . 2008. Chemotaxis of Escherichia coli to pyrimidines: a new role for the signal transducer tap. J Bacteriol 190:972–979. doi:10.1128/JB.01590-07 18065551 PMC2223585

[12] Liu X , Wood PL , Parales JV , Parales RE . 2009. Chemotaxis to pyrimidines and identification of a cytosine chemoreceptor in Pseudomonas putida. J Bacteriol 191:2909–2916. doi:10.1128/JB.01708-08 19251854 PMC2681813

[13] Martín-Mora D , Ortega A , Reyes-Darias JA , García V , López-Farfán D , Matilla MA , Krell T . 2016. Identification of a chemoreceptor in Pseudomonas aeruginosa that specifically mediates chemotaxis toward α-ketoglutarate. Front Microbiol 7:1937. doi:10.3389/fmicb.2016.01937 27965656 PMC5126104

[14] Alvarez-Ortega C , Harwood CS . 2007. Identification of a malate chemoreceptor in Pseudomonas aeruginosa by screening for chemotaxis defects in an energy taxis-deficient mutant. Appl Environ Microbiol 73:7793–7795. doi:10.1128/AEM.01898-07 17933940 PMC2168054

[15] Adler J , Hazelbauer GL , Dahl MM . 1973. Chemotaxis toward sugars in Escherichia coli. J Bacteriol 115:824–847. doi:10.1128/jb.115.3.824-847.1973 4580570 PMC246327

[16] Matilla MA , Velando F , Martín-Mora D , Monteagudo-Cascales E , Krell T . 2022. A catalogue of signal molecules that interact with sensor kinases, chemoreceptors and transcriptional regulators. FEMS Microbiol Rev 46:fuab043. doi:10.1093/femsre/fuab043 34424339

[17] Bi S , Jin F , Sourjik V . 2018. Inverted signaling by bacterial chemotaxis receptors. Nat Commun 9:2927. doi:10.1038/s41467-018-05335-w 30050034 PMC6062612

[18] Monteagudo-Cascales E , Martín-Mora D , Xu W , Sourjik V , Matilla MA , Ortega Á , Krell T . 2022. The pH robustness of bacterial sensing. mBio 13:e0165022. doi:10.1128/mbio.01650-22 36154178 PMC9600550

[19] Tohidifar P , Plutz MJ , Ordal GW , Rao CV . 2020. The mechanism of bidirectional pH taxis in Bacillus subtilis. J Bacteriol 202:e00491-19. doi:10.1128/JB.00491-19 31685537 PMC6989800

[20] Yang Y , Sourjik V . 2012. Opposite responses by different chemoreceptors set a tunable preference point in Escherichia coli pH taxis. Mol Microbiol 86:1482–1489. doi:10.1111/mmi.12070 23078189

[21] Yoney A , Salman H . 2015. Precision and variability in bacterial temperature sensing. Biophys J 108:2427–2436. doi:10.1016/j.bpj.2015.04.016 25992721 PMC4457042

[22] Paulick A , Jakovljevic V , Zhang S , Erickstad M , Groisman A , Meir Y , Ryu WS , Wingreen NS , Sourjik V . 2017. Mechanism of bidirectional thermotaxis in Escherichia coli. Elife 6:e26607. doi:10.7554/eLife.26607 28826491 PMC5578741

[23] Porter SL , Wadhams GH , Armitage JP . 2011. Signal processing in complex chemotaxis pathways. Nat Rev Microbiol 9:153–165. doi:10.1038/nrmicro2505 21283116

[24] Porter SL , Wadhams GH , Armitage JP . 2008. Rhodobacter sphaeroides: complexity in chemotactic signalling. Trends Microbiol 16:251–260. doi:10.1016/j.tim.2008.02.006 18440816

[25] Rao CV , Glekas GD , Ordal GW . 2008. The three adaptation systems of Bacillus subtilis chemotaxis. Trends Microbiol 16:480–487. doi:10.1016/j.tim.2008.07.003 18774298 PMC3532902

[26] Ortega DR , Fleetwood AD , Krell T , Harwood CS , Jensen GJ , Zhulin IB . 2017. Assigning chemoreceptors to chemosensory pathways in Pseudomonas aeruginosa. Proc Natl Acad Sci U S A 114:12809–12814. doi:10.1073/pnas.1708842114 29133402 PMC5715753

[27] Matilla MA , Martín-Mora D , Gavira JA , Krell T . 2021. Pseudomonas aeruginosa as a model to study chemosensory pathway signaling. Microbiol Mol Biol Rev 85:e00151-20. doi:10.1128/MMBR.00151-20 33441490 PMC7849354

[28] Kühn MJ , Talà L , Inclan YF , Patino R , Pierrat X , Vos I , Al-Mayyah Z , Macmillan H , Negrete J , Engel JN , Persat A . 2021. Mechanotaxis directs Pseudomonas aeruginosa twitching motility. Proc Natl Acad Sci U S A 118:e2101759118. doi:10.1073/pnas.2101759118 34301869 PMC8325320

[29] O’Neal L , Baraquet C , Suo Z , Dreifus JE , Peng Y , Raivio TL , Wozniak DJ , Harwood CS , Parsek MR . 2022. The Wsp system of Pseudomonas aeruginosa links surface sensing and cell envelope stress. Proc Natl Acad Sci U S A 119:e2117633119. doi:10.1073/pnas.2117633119 35476526 PMC9170161

[30] Kühn MJ , Macmillan H , Talà L , Inclan Y , Patino R , Pierrat X , Al-Mayyah Z , Engel JN , Persat A . 2023. Two antagonistic response regulators control Pseudomonas aeruginosa polarization during mechanotaxis. EMBO J 42:e112165. doi:10.15252/embj.2022112165 36795017 PMC10519157

[31] Wu H , Kato J , Kuroda A , Ikeda T , Takiguchi N , Ohtake H . 2000. Identification and characterization of two chemotactic transducers for inorganic phosphate in Pseudomonas aeruginosa. J Bacteriol 182:3400–3404. doi:10.1128/JB.182.12.3400-3404.2000 10852870 PMC101905

[32] Rico-Jiménez M , Reyes-Darias JA , Ortega Á , Díez Peña AI , Morel B , Krell T . 2016. Two different mechanisms mediate chemotaxis to inorganic phosphate in Pseudomonas aeruginosa. Sci Rep 6:28967. doi:10.1038/srep28967 27353565 PMC4926252

[33] Martín-Mora D , Ortega Á , Matilla MA , Martínez-Rodríguez S , Gavira JA , Krell T . 2019. The molecular mechanism of nitrate chemotaxis via direct ligand binding to the Pilj domain of MCPN. MBio 10:e02334-18. doi:10.1128/mBio.02334-18 30782655 PMC6381276

[34] Reyes-Darias JA , García V , Rico-Jiménez M , Corral-Lugo A , Lesouhaitier O , Juárez-Hernández D , Yang Y , Bi S , Feuilloley M , Muñoz-Rojas J , Sourjik V , Krell T . 2015. Specific gamma-aminobutyrate chemotaxis in pseudomonads with different lifestyle. Mol Microbiol 97:488–501. doi:10.1111/mmi.13045 25921834

[35] Rico-Jiménez M , Muñoz-Martínez F , García-Fontana C , Fernandez M , Morel B , Ortega A , Ramos JL , Krell T . 2013. Paralogous chemoreceptors mediate chemotaxis towards protein amino acids and the non-protein amino acid gamma-aminobutyrate (GABA). Mol Microbiol 88:1230–1243. doi:10.1111/mmi.12255 23650915

[36] Reyes-Darias JA , Yang Y , Sourjik V , Krell T . 2015. Correlation between signal input and output in PctA and PctB amino acid chemoreceptor of Pseudomonas aeruginosa. Mol Microbiol 96:513–525. doi:10.1111/mmi.12953 25641105

[37] Dhodary B , Sampedro I , Behroozian S , Borza V , Her S , Hill JE . 2022. The arginine catabolism-derived amino acid l-ornithine is a chemoattractant for Pseudomonas aeruginosa. Microorganisms 10:264. doi:10.3390/microorganisms10020264 35208720 PMC8875649

[38] Fernández M , Ortega Á , Rico-Jiménez M , Martín-Mora D , Daddaoua A , Matilla MA , Krell T . 2018. High-throughput screening to identify chemoreceptor ligands. Methods Mol Biol 1729:291–301. doi:10.1007/978-1-4939-7577-8_23 29429099

[39] McKellar JLO , Minnell JJ , Gerth ML . 2015. A high‐throughput screen for ligand binding reveals the specificities of three amino acid chemoreceptors from Pseudomonas syringae pv. actinidiae. Mol Microbiol 96:694–707. doi:10.1111/mmi.12964 25656450

[40] Matilla MA , Martín-Mora D , Krell T . 2020. The use of isothermal titration calorimetry to unravel chemotactic signalling mechanisms. Environ Microbiol 22:3005–3019. doi:10.1111/1462-2920.15035 32329116

[41] Fernández M , Morel B , Corral-Lugo A , Rico-Jiménez M , Martín-Mora D , López-Farfán D , Reyes-Darias JA , Matilla MA , Ortega Á , Krell T . 2016. Identification of ligands for bacterial sensor proteins. Curr Genet 62:143–147. doi:10.1007/s00294-015-0528-4 26511375

[42] Bi S , Pollard AM , Yang Y , Jin F , Sourjik V . 2016. Engineering hybrid chemotaxis receptors in bacteria. ACS Synth Biol 5:989–1001. doi:10.1021/acssynbio.6b00053 27285081

[43] Lehning CE , Heidelberger JB , Reinhard J , Nørholm MHH , Draheim RR . 2017. A modular high-throughput in vivo screening platform based on chimeric bacterial receptors. ACS Synth Biol 6:1315–1326. doi:10.1021/acssynbio.6b00288 28372360

[44] Fernández M , Matilla MA , Ortega Á , Krell T . 2017. Metabolic value chemoattractants are preferentially recognized at broad ligand range chemoreceptor of Pseudomonas putida KT2440. Front Microbiol 8:990. doi:10.3389/fmicb.2017.00990 28620365 PMC5449446

[45] Dash SS , Sailaja NS , Gummadi SN . 2008. Chemotaxis of Pseudomonas sp. to caffeine and related methylxanthines. J Basic Microbiol 48:130–134. doi:10.1002/jobm.200700273 18383225

[46] Fernández M , Corral-Lugo A , Krell T . 2018. The plant compound rosmarinic acid induces a broad quorum sensing response in Pseudomonas aeruginosa PAO1. Environ Microbiol 20:4230–4244. doi:10.1111/1462-2920.14301 30051572

[47] Rico-Jiménez M , Roca A , Krell T , Matilla MA . 2022. A bacterial chemoreceptor that mediates chemotaxis to two different plant hormones. Environ Microbiol 24:3580–3597. doi:10.1111/1462-2920.15920 35088505 PMC9543091

[48] Sampedro I , Parales RE , Krell T , Hill JE . 2015. Pseudomonas chemotaxis. FEMS Microbiol Rev 39:17–46. doi:10.1111/1574-6976.12081 25100612

[49] Gavira JA , Gumerov VM , Rico-Jiménez M , Petukh M , Upadhyay AA , Ortega A , Matilla MA , Zhulin IB , Krell T . 2020. How bacterial chemoreceptors evolve novel ligand specificities. mBio 11:e03066-19. doi:10.1128/mBio.03066-19 31964737 PMC6974571

[50] Fernández M , Rico-Jiménez M , Ortega Á , Daddaoua A , García García AI , Martín-Mora D , Torres NM , Tajuelo A , Matilla MA , Krell T . 2019. Determination of ligand profiles for Pseudomonas aeruginosa solute binding proteins. Int J Mol Sci 20:5156. doi:10.3390/ijms20205156 31627455 PMC6829864

[51] Parales RE , Nesteryuk V , Hughes JG , Luu RA , Ditty JL . 2014. Cytosine chemoreceptor McpC in Pseudomonas putida F1 also detects nicotinic acid. Microbiology 160:2661–2669. doi:10.1099/mic.0.081968-0 25294107 PMC4811638

[52] Hida A , Oku S , Miura M , Matsuda H , Tajima T , Kato J . 2020. Characterization of methyl-accepting chemotaxis proteins (MCPs) for amino acids in plant-growth-promoting rhizobacterium Pseudomonas protegens CHA0 and enhancement of amino acid chemotaxis by MCP genes overexpression. Biosci Biotechnol Biochem 84:1948–1957. doi:10.1080/09168451.2020.1780112 32538292

[53] Sourjik V , Vaknin A , Shimizu TS , Berg HC . 2007. In vivo measurement by FRET of pathway activity in bacterial chemotaxis. Methods Enzymol 423:365–391. doi:10.1016/S0076-6879(07)23017-4 17609141

[54] Somavanshi R , Ghosh B , Sourjik V . 2016. Sugar influx sensing by the phosphotransferase system of Escherichia coli. PLoS Biol 14:e2000074. doi:10.1371/journal.pbio.2000074 27557415 PMC4996493

[55] Lux R , Jahreis K , Bettenbrock K , Parkinson JS , Lengeler JW . 1995. Coupling the phosphotransferase system and the methyl-accepting chemotaxis protein-dependent chemotaxis signaling pathways of Escherichia coli. Proc Natl Acad Sci U S A 92:11583–11587. doi:10.1073/pnas.92.25.11583 8524808 PMC40446

[56] Neumann S , Grosse K , Sourjik V . 2012. Chemotactic signaling via carbohydrate phosphotransferase systems in Escherichia coli. Proc Natl Acad Sci U S A 109:12159–12164. doi:10.1073/pnas.1205307109 22778402 PMC3409764

[57] Krikos A , Conley MP , Boyd A , Berg HC , Simon MI . 1985. Chimeric chemosensory transducers of Escherichia coli. Proc Natl Acad Sci U S A 82:1326–1330. doi:10.1073/pnas.82.5.1326 3883356 PMC397253

[58] Doeun D , Davaatseren M , Chung M-S . 2017. Biogenic amines in foods. Food Sci Biotechnol 26:1463–1474. doi:10.1007/s10068-017-0239-3 30263683 PMC6049710

[59] Pugin B , Barcik W , Westermann P , Heider A , Wawrzyniak M , Hellings P , Akdis CA , O’Mahony L . 2017. A wide diversity of bacteria from the human gut produces and degrades biogenic amines. Microb Ecol Health Dis 28:1353881. doi:10.1080/16512235.2017.1353881 28959180 PMC5614385

[60] Galperin MY . 2018. What bacteria want. Environ Microbiol 20:4221–4229. doi:10.1111/1462-2920.14398 30187651 PMC7020242

[61] Matilla MA , Monteagudo-Cascales E , Krell T . 2023. Advances in the identification of signals and novel sensing mechanisms for signal transduction systems. Environ Microbiol 25:79–86. doi:10.1111/1462-2920.16142 35896893

[62] Upadhyay AA , Fleetwood AD , Adebali O , Finn RD , Zhulin IB . 2016. Cache domains that are homologous to, but different from PAS domains comprise the largest superfamily of extracellular sensors in prokaryotes. PLoS Comput Biol 12:e1004862. doi:10.1371/journal.pcbi.1004862 27049771 PMC4822843

[63] Ravikumar S , David Y , Park SJ , Choi J-I . 2018. A chimeric two-component regulatory system-based Escherichia coli biosensor engineered to detect glutamate. Appl Biochem Biotechnol 186:335–349. doi:10.1007/s12010-018-2746-y 29611135

[64] Kishii R , Falzon L , Yoshida T , Kobayashi H , Inouye M . 2007. Structural and functional studies of the HAMP domain of EnvZ, an osmosensing transmembrane histidine kinase in Escherichia coli. J Biol Chem 282:26401–26408. doi:10.1074/jbc.M701342200 17635923

[65] Samant S , Lee H , Ghassemi M , Chen J , Cook JL , Mankin AS , Neyfakh AA . 2008. Nucleotide biosynthesis is critical for growth of bacteria in human blood. PLoS Pathog 4:e37. doi:10.1371/journal.ppat.0040037 18282099 PMC2242838

[66] Turner KH , Wessel AK , Palmer GC , Murray JL , Whiteley M . 2015. Essential genome of Pseudomonas aeruginosa in cystic fibrosis sputum. Proc Natl Acad Sci U S A 112:4110–4115. doi:10.1073/pnas.1419677112 25775563 PMC4386324

[67] Al Mahmud H , Baishya J , Wakeman CA . 2021. Interspecies metabolic complementation in cystic fibrosis pathogens via purine exchange. Pathogens 10:146. doi:10.3390/pathogens10020146 33535659 PMC7912780

[68] Rahman H , King RM , Shewell LK , Semchenko EA , Hartley-Tassell LE , Wilson JC , Day CJ , Korolik V . 2014. Characterisation of a multi-ligand binding chemoreceptor CcmL (Tlp3) of Campylobacter jejuni. PLoS Pathog 10:e1003822. doi:10.1371/journal.ppat.1003822 24391495 PMC3879368

[69] Fernández M , Morel B , Corral-Lugo A , Krell T . 2016. Identification of a chemoreceptor that specifically mediates chemotaxis toward metabolizable purine derivatives. Mol Microbiol 99:34–42. doi:10.1111/mmi.13215 26355499

[70] Bernier SP , Ha D-G , Khan W , Merritt JH , O’Toole GA . 2011. Modulation of Pseudomonas aeruginosa surface-associated group behaviors by individual amino acids through c-di-GMP signaling. Res Microbiol 162:680–688. doi:10.1016/j.resmic.2011.04.014 21554951 PMC3716369

[71] Maarsingh H , Pera T , Meurs H . 2008. Arginase and pulmonary diseases. Naunyn Schmiedebergs Arch Pharmacol 378:171–184. doi:10.1007/s00210-008-0286-7 18437360 PMC2493601

[72] Schwarzer C , Fischer H , Machen TE . 2016. Chemotaxis and binding of Pseudomonas aeruginosa to scratch-wounded human cystic fibrosis airway epithelial cells. PLoS One 11:e0150109. doi:10.1371/journal.pone.0150109 27031335 PMC4816407

[73] O’Donnell MP , Fox BW , Chao P-H , Schroeder FC , Sengupta P . 2020. A neurotransmitter produced by gut bacteria modulates host sensory behaviour. Nature 583:415–420. doi:10.1038/s41586-020-2395-5 32555456 PMC7853625

[74] Gumerov VM , Andrianova EP , Matilla MA , Page KM , Monteagudo-Cascales E , Dolphin AC , Krell T , Zhulin IB . 2022. Amino acid sensor conserved from bacteria to humans. Proc Natl Acad Sci U S A 119:e2110415119. doi:10.1073/pnas.2110415119 35238638 PMC8915833

[75] Choi K-H , Kumar A , Schweizer HP . 2006. A 10-min method for preparation of highly electrocompetent Pseudomonas aeruginosa cells: application for DNA fragment transfer between chromosomes and plasmid transformation. J Microbiol Methods 64:391–397. doi:10.1016/j.mimet.2005.06.001 15987659

[76] Abril MA , Michan C , Timmis KN , Ramos JL . 1989. Regulator and enzyme specificities of the TOL plasmid-encoded upper pathway for degradation of aromatic hydrocarbons and expansion of the substrate range of the pathway. J Bacteriol 171:6782–6790. doi:10.1128/jb.171.12.6782-6790.1989 2687253 PMC210577

[77] Stukenberg D , Hensel T , Hoff J , Daniel B , Inckemann R , Tedeschi JN , Nousch F , Fritz G . 2021. The Marburg collection: a golden gate DNA assembly framework for synthetic biology applications in Vibrio natriegens ACS Synth Biol 10:3236. doi:10.1021/acssynbio.1c00497 34255476

[78] Sourjik V , Berg HC . 2002. Receptor sensitivity in bacterial chemotaxis. Proc Natl Acad Sci U S A 99:123–127. doi:10.1073/pnas.011589998 11742065 PMC117525

[79] Si G , Yang W , Bi S , Luo C , Ouyang Q . 2012. A parallel diffusion-based microfluidic device for bacterial chemotaxis analysis. Lab Chip 12:1389–1394. doi:10.1039/c2lc21219f 22361931

[80] Zhao H , Piszczek G , Schuck P . 2015. SEDPHAT--a platform for global ITC analysis and global multi-method analysis of molecular interactions. Methods 76:137–148. doi:10.1016/j.ymeth.2014.11.012 25477226 PMC4380758

