# Supplementary material for: Systematic mapping of chemoreceptor specificities for Pseudomonas aeruginosa
Source: mBio. 2023 Oct 4;14(5):e02099-23. doi: 10.1128/mbio.02099-23 (PMC10653921; doi:10.1128/mbio.02099-23)
Supplement: Supplemental Material — Fig. S1 to S6; Tables S1 to S4 [file mbio.02099-23-s0001.pdf]

Supplemental material

to

Systematic mapping of chemoreceptor specificities for *Pseudomonas aeruginosa*

by

Wenhao Xu, Jean Paul Cerna-Vargas, Ana Tajuelo, Andrea Lozano-Montoya, Melissa Kivoloka, Nicolas Krink, Elizabet Monteagudo-Cascales, Miguel A. Matilla, Tino Krell, Victor Sourjik

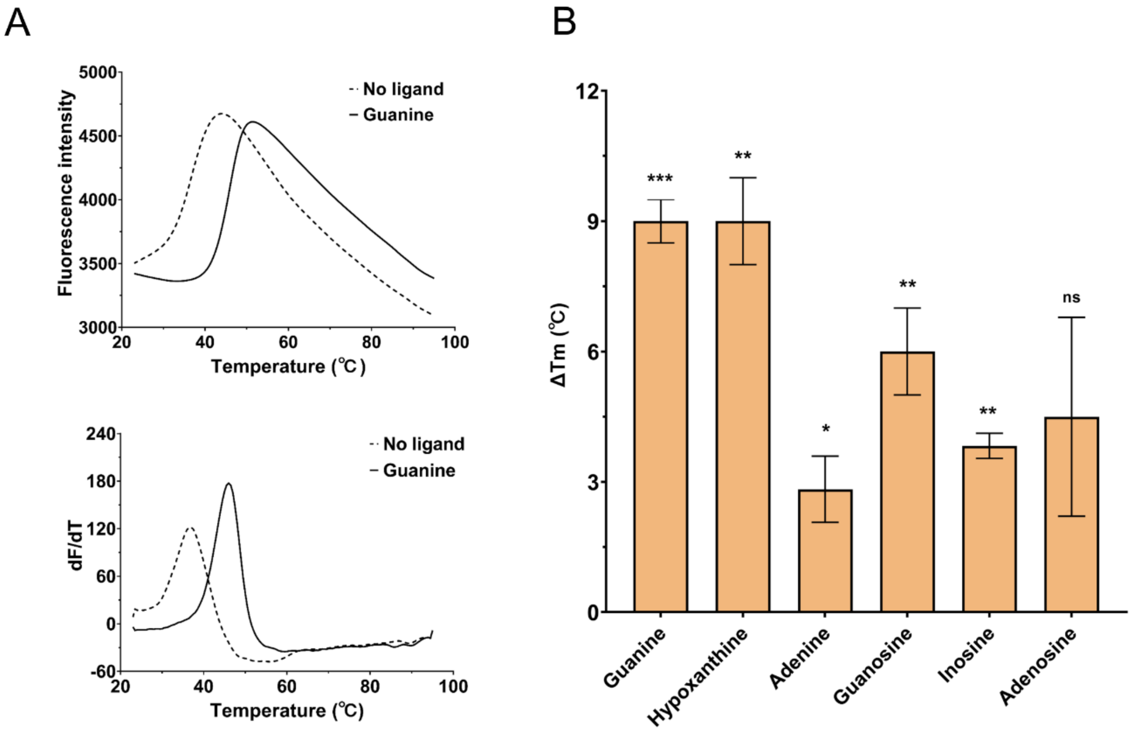

Fig. S1 Thermal shift assay for purified PctP-LBD in the presence of purine derivatives. (A) Thermal unfolding of PctP-LBD in the absence or in the presence of 2 mM guanine (upper panel: raw data; lower panel: their first derivative). (B) Increases in the midpoint temperature of protein unfolding transition (Tm) induced by indicated ligands. Data shown are the means and standard deviations from three biological replicates conducted in triplicate. Significance of difference from zero (no thermal shift), assessed by one sample *t*-test, is indicated by asterisks (ns: non-significant, *$p \leq 0.05$, **$p \leq 0.01$, ***$p \leq 0.001$).

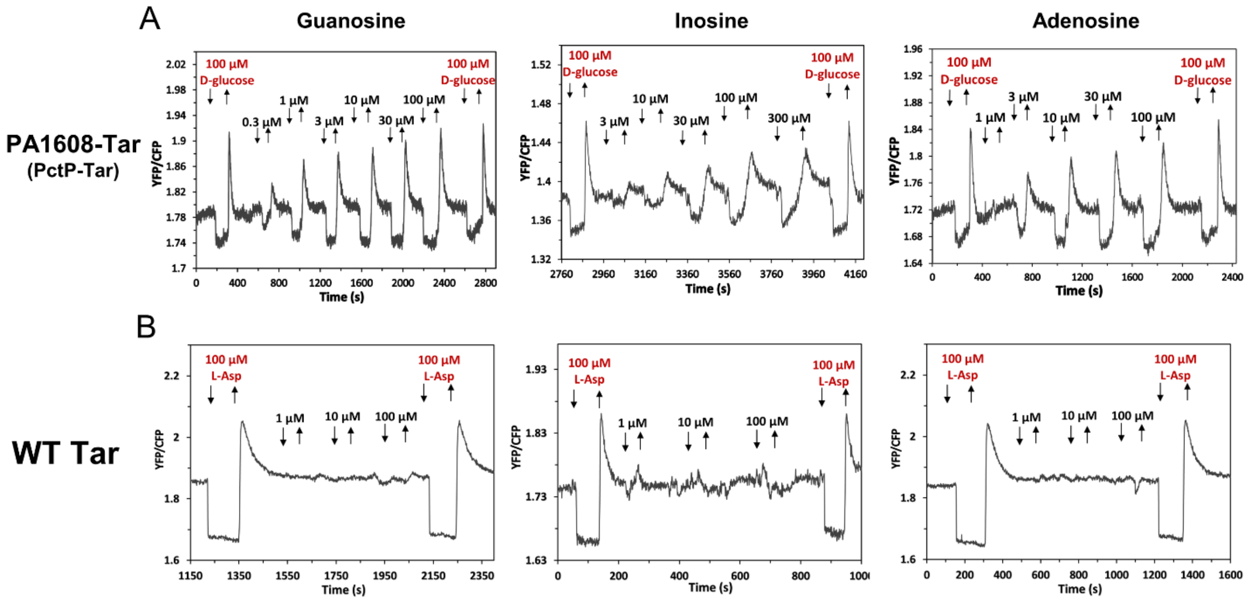

Fig. S2 PctP-Tar hybrid mediates specific FRET responses to purine nucleosides. FRET measurements for *E. coli* cells expressing PctP-Tar hybrid (A) or Tar (B) as a sole receptor, responding to the indicated concentrations of guanosine, inosine or adenosine. The activity of PctP-Tar was confirmed using D-glucose. Similarly, L-aspartate (L-Asp) was used as a positive ligand for Tar.

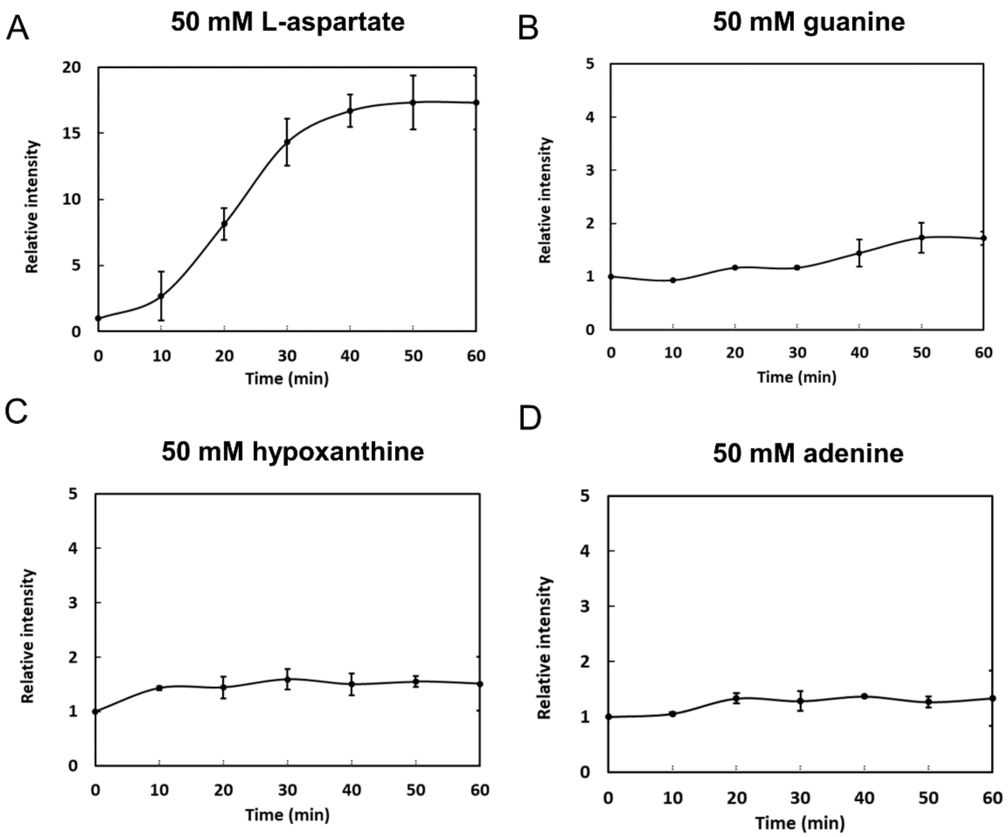

Fig. S3 Control microfluidic assays for responses of *E. coli* expressing Tar to purines. Experiments were performed as in Fig. 4C, with L-Asp used as a positive control for Tar.

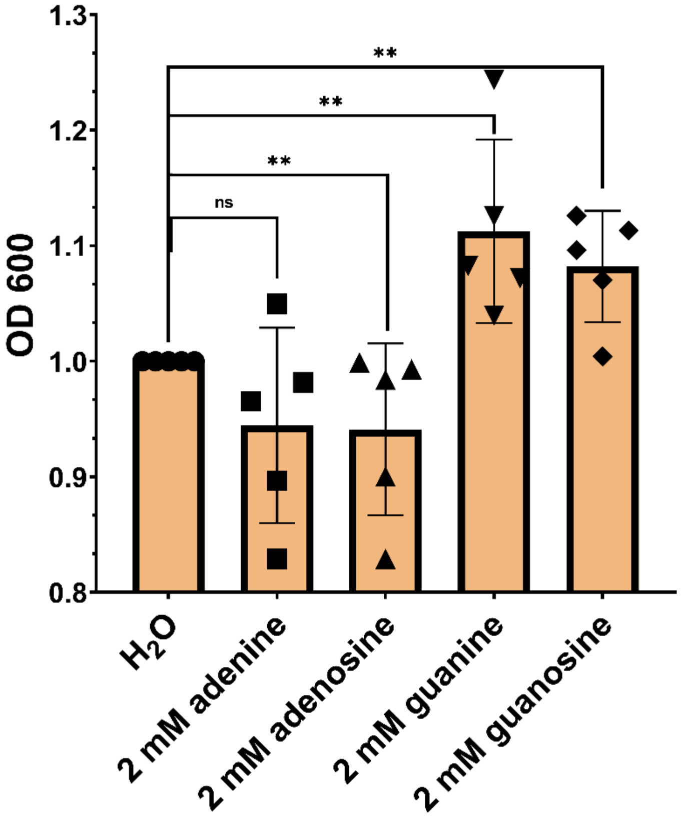

Fig. S4 Effects of purine derivatives on the growth of *P. aeruginosa* PAO1. PAO1 was cultured at 37°C in LB containing 2 mM of the indicated compounds, and the same volume of H_2O was added to the culture as control. Optical density of cultures was measured at 600 nm after growing 12 hours with shaking in the microplate reader. For each experiment, the OD600 was normalized to the OD600 of the control culture in LB. The means and standard deviations of five biological replicates each conducted in triplicate were shown. Error bars indicated the mean ± standard deviations. Significance of differences, assessed using a paired *t*-test, is indicated by asterisks (ns: non-significant, $^{**}p \leqslant 0.01$).

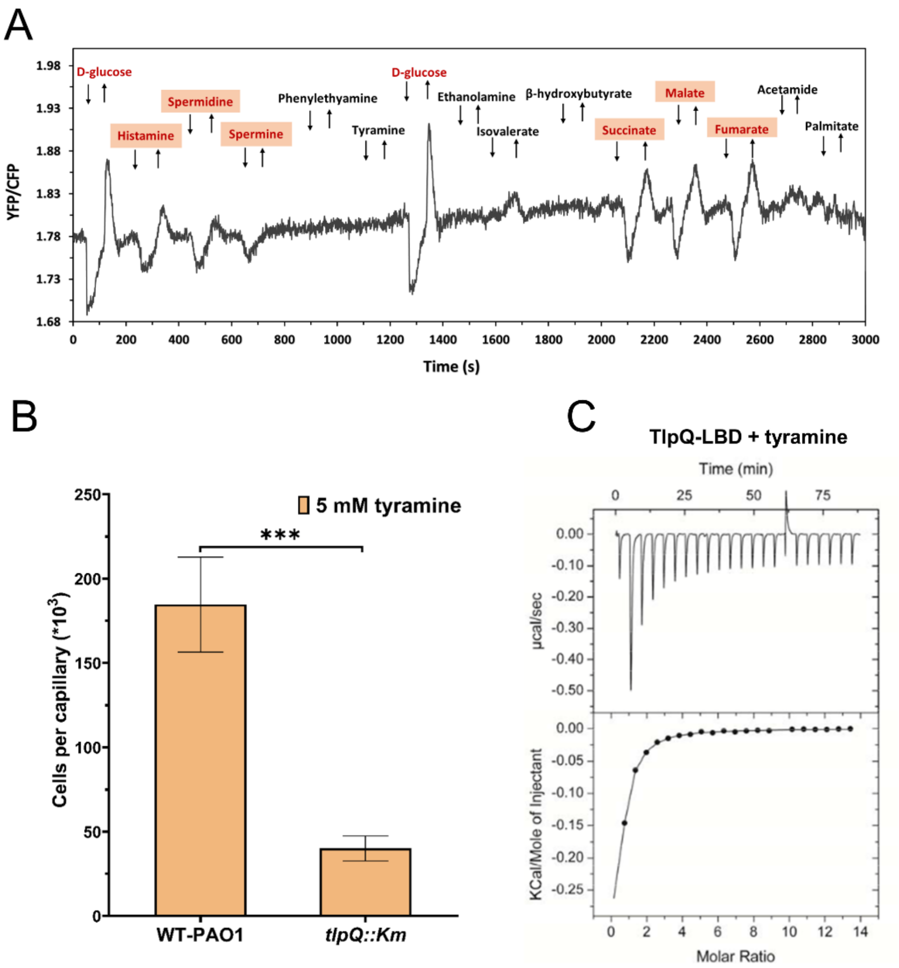

Fig. S5 Characterization of TlpQ as a chemoreceptor for tyramine. (A) Screening of unassigned potential ligands for TlpQ-Tar using FRET. FRET measurement was performed for *E. coli* cells expressing TlpQ-Tar as the sole receptor, exposed to a stepwise addition (down arrow) and subsequent removal (up arrow) of 100 µM D-glucose (positive ligand for chimeras), three known ligands (histamine, spermine and spermidine) and other chemical compounds. The chemoattractants are shown in red. (B) Capillary chemotaxis assays of *tlpQ* deficient strain and WT-PAO1 (WT-Hiroshima) to 5 mM tyramine. Data are shown as the means and standard deviations from three biological replicates conducted in triplicate. Significance of difference, assessed using a paired *t*-test, is indicated by asterisks (***$p \leq 0.001$). (C) Microcalorimetric studies confirming the binding of tyramine to TlpQ-LBD. The upper panel shows raw titration data, whereas the lower panel shows the fit of the integrated concentration-normalized and dilution-heat corrected raw data using the single-site binding model. Further experimental details are provided in Table S4.

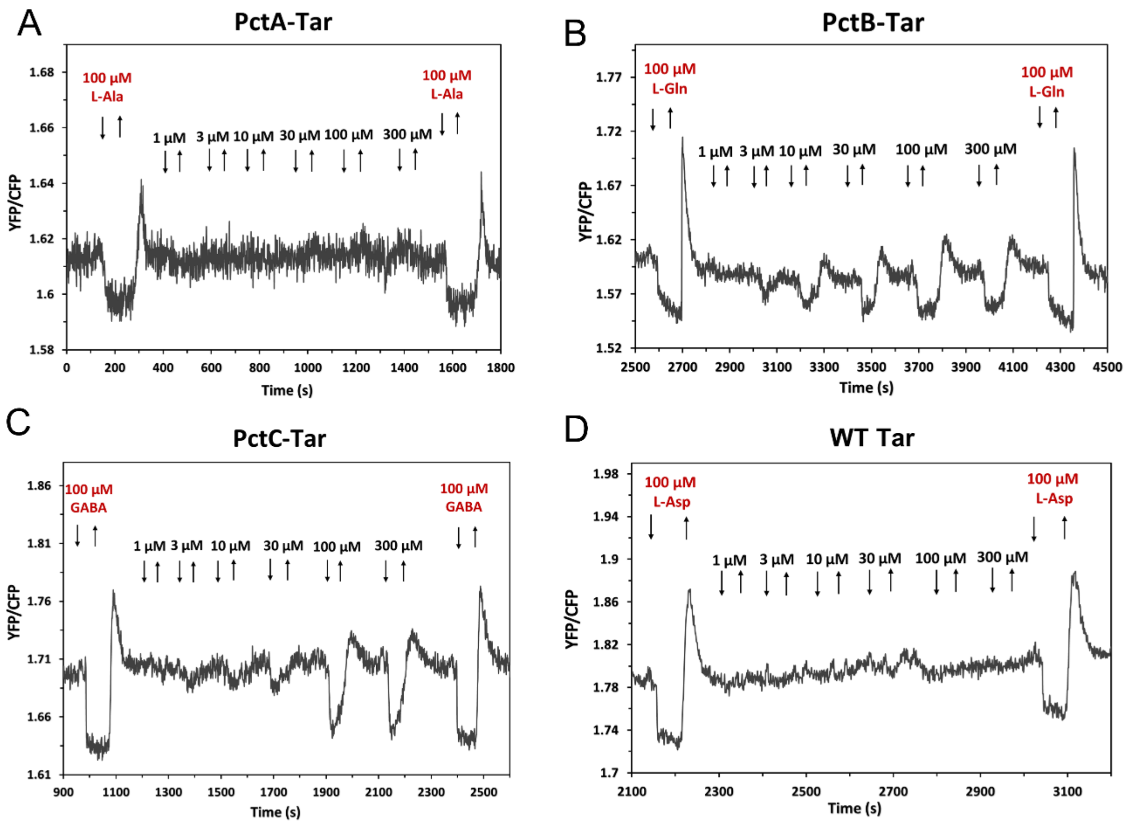

Fig. S6 PctB and PctC mediate responses to histamine. FRET measurements of responses mediated by PctA-Tar (A), PctB-Tar (B), PctC-Tar (C) or Tar (D) as a sole receptor to indicated concentrations of histamine.

Table S1 Growth of *P. aeruginosa* PAO1 in M9 minimal medium supplemented with each of the compounds present in the Biolog plates PM1, PM2A and PM3B as nitrogen source (in nitrogen-free medium) or carbon source (in carbon-free medium). A total of 202 compounds supported bacterial growth at different levels. Compounds were divided into different groups according to the magnitude of growth. The 29 highlighted compounds were further studied using quantitative capillary chemotaxis assays (Fig. 1).

**No.**	**High bacterial growth (OD_{600nm}>0.25)**	**Medium bacterial growth (0.25>OD_{600nm}>0.1)**	**Low bacterial growth (0.1<OD_{600nm}<0.05)**
1	p-Hydroxy Phenyl Acetic Acid	Citric Acid	Mucic Acid
2	D, L-Malic Acid	L-Malic Acid	**Tricarballylic Acid**
3	**D-Gluconic Acid**	**Fumaric Acid**	D-Glucosaminic Acid
4	L-Asn	**Propionic Acid**	Acetoacetic Acid
5	L-Gln	α-Keto-Glutaric Acid	D-Galactonic Acid-γ-Lactone
6	L-Ala	Bromo Succinic Acid	Glycolic Acid
7	L-Pro	**Pyruvic Acid**	D-Galacturonic Acid
8	Gly-L-Pro	Acetic Acid	**Succinic Acid**
9	Tyramine	α-Hydroxy Butyric Acid	**Formic Acid**
10	D-Fructose	L-Lactic Acid	m-Hydroxy Phenyl Acetic Acid
11	**D-Glucose**	α-Hydroxy Glutaric Acid-γ-Lactone	D-Glucuronic Acid
12	N-Acetyl-Dglucosamine	α-Keto-Butyric Acid	m-Tartaric Acid
13	**D-Mannitol**	D-Malic Acid	Mono Methyl Succinate
14	Glycerol	Methyl Pyruvate	D-Ser
15	**Itaconic Acid**	L-Ser	**2-Phenylethylamine**
16	**5-Amino Valeric Acid**	L-Thr	Ammonia
17	Capric Acid	L-Asp	α-Methyl-D-Glucoside
18	Sebacic Acid	L-Glu	D-Fructose-6-Phosphate
19	γ-Amino Butyric Acid	D-Thr	D-Psicose
20	4-Hydroxy Benzoic Acid	D-Ala	D-Glucose-6-Phosphate
21	**ß-Hydroxy Butyric Acid**	D-Asp	**D-Maltose**
22	Quinic Acid	Gly-L-Glu	L-Fucose
23	γ -Hydroxy Butyric Acid	2-Aminoethanol	D-Glucose-1-Phosphate
24	Caproic Acid	**D-Trehalose**	**D-Xylose**
25	N-Acetyl-L-Glutamic Acid	Maltotriose	D-Cellobiose
26	L-PyroglutamicAcid	D-Melibiose	L-Arabinose
27	**L-Orn**	α-Methyl-D-galactoside	N-Acetyl-ß-D-Mannosamine
28	Hydroxy-Lproline	Sucrose	Adonitol
29	L-Ile	Lactulose	D, L-α-Glycerol- Phosphate
30	L-Arg	α-D-Lactose	Dulcitol

31	D, L-Octopamine	**D-Ribose**	myo-Inositol
32	Putrescine	2-Deoxy Adenosine	D-Sorbitol
33	**Glucosamine**	Tween 20	Thymidine
34	Uric Acid	Tween 40	α- Keto-Valeric Acid
35	L-His	Tween 80	2- Hydroxy Benzoic Acid
36	L-Trp	**Butyric Acid**	Oxalic Acid
37	L-Val	Citramalic Acid	ß-Methyl-D-Glucuronic Acid
38	Ala-His	Sorbic Acid	Melibionic Acid
39	Ala-Gln	Succinamic Acid	L-Tartaric Acid
40	Ala-Glu	Malonic Acid	Citraconic Acid
41	Gly-Asn	D, L-Carnitine	N-Acetyl-Neuraminic Acid
42	Gly-Gln	Laminarin	**D-Tartaric Acid**
43	Ala-Asp	Turanose	L-homoserine
44	**Ethanolamine**	Xylitol	L- Leu
45	Agmatine	D-Arabitol	L-Met
46	Histamine	γ- Amino-N-Butyric Acid	L-Alaninamide
47	Allantoin	D, L-α-Amino-N-Butyric Acid	L-Phe-Ala
48	Urea	α-Amino-N-Valeric Acid	Gly-Met
49	**Acetamide**	ε-Amino-N-Caproic Acid	3-0-ß-D-Galactopyranosyl-D-Arabinose
50	**Adenosine**	Parabanic Acid	**Salicylic acid**
51	**Inosine**	δ-Amino-N-Valeric Acid	D-Raffinose
52	Adenine	N-Acetyl-L-Glutamic Acid	ß-Methyl-D-Xyloside
53	Guanosine	N-Phthaloyl-L-Glutamic Acid	α-Methyl-D-Mannoside
54	**Cytosine**	D-Glu	Isoatinose
55	Cytidine	L-Glu	D-Tagatose
56	Thymine	L-Lys	Gentiobiose
57	**Uridine**	L-Tyr	D-Fucose
58		D-Lys	3-Methyl Glucose
59		D-Asn	D-Ribono-1,4-Lactone
60		L-Gly	L-Glucose
61		Ala-Gly	D-Melezitose
62		Ala-Leu	ß-D-Allose
63		Uracil	D-Arabinose
64		Glucuronamide	ß-Methyl-D-Galactoside
65		N-Acetyl-D-Glucosamine	Amygdalin

66		Nitrite	Sedoheptulosan
67		Nitrate	N-Acetyl-D-Galactosamine
68		Xanthosine	N-Acetyl-D-Glucosaminitol
69		Xanthine	i-Erythritol
70			Lactitol
71			Maltitol
72			D-Val
73			**Guanine**
74			L- Cys
75			L-Citrulline
76			Met-Ala

Table S2 Summary of FRET responses of 16 hybrid chemoreceptors to tested ligands. All ligand solutions were at 100 µM concentration. The attractant responses are shown in red; and the repellent responses are shown in green; "CR": these responses might be mediated by the cytoplasmic portion of Tar; "--": no specific responses. The numbers are the normalized magnitude of corresponding compound's response to the magnitude of characterized ligand's response or D-glucose's response, whichever is greater. Data are shown as the means and standard deviations of results from at least three biological replicates.

Categories	Compounds	Tar	PctA	PctB	PctC	PctD	CtpM	PA2867	PctP	TlpQ	PA1646	PA4844	PA2573	PA1251	PA2788	PA2920	McpK	PA4915
Positive control	D-glucose	CR	CR	CR	CR	CR	CR	CR	CR	CR	CR	CR	CR	CR	CR	CR	CR	CR
Organic acids	Pyruvate		--	--	--	--	--	--	--	--	--	--	--	--	--	--	--	--
	Itaconate	--	--	--	--	--	--	--	--	--	--	--	--	--	--	--	--	--
	Formate	--	--	--	--	--	--	--	--	--	--	--	--	--	--	--	--	--
	Fumarate	CR	--	--		--	--	--	--	--	--	--	--	--	--	--	--	--
	Propionate	--	--	--	--	--	--	--	--	--	--	--	--	--	--	--	--	--
	Succinate	CR	--	--	--	--	--	--	--	--	--	--	--	--	--	--	--	--
	Butyrate	--	--	--	--	--	--	--	--	--	--	--	--	--	--	--	--	--
	Glutarate	--	--	--	--	--	--	--	--	--	--	--	--	--	--	--	--	--
	D-gluconate	--	--	--	--	--	--	--	--	--	--	--	--	--	--	--	--	--
	D-tartrate	CR	--	--	--	--	--	--	--	--	--	--	--	--	--	--	--	--
	Salicylate	--	--	--	--	--	--	2.65 ± 0.37[a]	--	--	--	--	--	--	--	--	--	--
	Nicotinate	--		--	--		--	--	--	--	--	--	--	--	--	--	--	--
	Isovalerate	--	--	--	--	--	--	--		--	--	--	--	--	--	--	--	--
	5-Aminovalerate	--	--	--	1.01 ± 0.09	--	--	--	--	--	--	--	--	--	--	--	--	--
	Methyl 4-aminobutyrate	--	--	--	0.97 ± 0.07	--	--	--	--	--	--	--	--	--	--	--	--	--
	β-hydroxybutyrate	--	--	--	--	--	--	--	--	--	--	--	--	--	--	--	--	--
	Palmitate	--	--	--	--	--	--	--	--	--	--	--	--	--	--	--	--	--
	Indoleacetate	--	--	--	--	--	--	--	--	--	--	--	--	--	--	--	--	--
Sugars	D-fructose	CR	--	--	--	--	--	--	--	--	--	--	--	--	--	--	--	--
	D-mannitol	CR	--	--	--	--	--		--	--	--	--	--	--	--	--	--	--
	D-xylose	--	--	--	--	--	--	--	--	--	--	--	--	--	--	--	--	--
	D-maltose	0.73 ± 0.13	--	--	--	--	--	--	--	--	--	--	--	--	--	--	--	--

	**D-trehalose**	CR	--	--	--	--	--	--	--	--	--	--	--	--	--	--	--	--
	**Glucosamine**	CR	--	--	--	--	--	--	--	--	--	--	--	--	--	--	--	--
**Nucleic acid derivatives**	**Uridine**	CR	--	--	--	--	--	--	--	--	--	--	--	--	--	--	--	--
	**D-ribose**	--	--	--	--	--	--	--	--	--	--	--	--	--	--	--	--	--
	**Cytosine**	--	--	--	--	--	--	--	--	--	--	--	--	--	--	--	--	--
	**Guanine**		--		--	--	--	--	1.22 ± 0.07	--	--	--	--	--	--	--	--	--
	**Inosine**	--	--	--	--	--	--	--	1.03 ± 0.08	--	--	--	--	--	--	--	--	--
	**Adenine**	--	--	--	--	--	--	--	0.79 ± 0.05	--	--	--	--	--	--	--	--	--
**Others**	**L-ornithine**	--	1.16 ± 0.10	1.30 ± 0.13	--	--	--	--	--	--	--	--	--	--	--	--	--	--
	**Ethanolamine**	--	--	--	--	--	--	--	--	--	--	--	--	--	--	--	--	--
	**2-Phenylethylamine**	--	--	--	--	--	--	--		--	--	--	--	--	--	--	--	--
	**Acetamide**	--	--	--	--	--	--	--	--	--	--	--	--	--	--	--	--	--
	**Caffeine**	--	--	--	--	--	--	--	--	--	--	--	--	--	--	--	--	--
**pH**	**pH6.0**	1.18 ± 0.16	0.71 ± 0.16	--	1.06 ± 0.09	--	--	--	1.92 ± 0.17	1.36 ± 0.20	--	--	--	--	--	--	--	--
	**pH8.0**	0.96 ± 0.11	1.02 ± 0.22	--	2.31 ± 0.17	--	--	--	0.99 ± 0.11	0.57 ± 0.12	--	--	--	--	--	--	--	--

[a]*E. coli* cells expressing PA2867-Tar showed a repellent response to salicylate. However, salicylate binding to PA2867 could not be confirmed because of the failure to purify PA2867-LBD as a stable protein, and characterization of this putative ligand was not further pursued.

Table S3 Strains, plasmids and oligonucleotides used in this study.

Strains and plasmids	Genotype or relevant characteristics[a]	Reference
**Strains**		
Escherichia coli BL21(DE3)	F^- *ompT gal dcm lon* $hsdS_B(r_B^- m_B^-)$ λ (DE3 [*lacI lacUV5-T7p07 ind1 sam7 nin5*]) [$malB^+$] $_{K-12}$(λ^S)	(1)
E. coli DH5α	F^- *endA1 glnV44 thi-1 recA1 relA1 gyrA96 deoR nupG purB20* φ80d*lacZ*ΔM15 Δ(*lacZYA-argF*) U169, hsdR17($r_K^- m_K^+$), λ^-	(2)
E. coli UU1250	Derivative of RP437; Δ*aer*Δ*tsr*Δ(*tar-tap*) Δ*trg*	(3)
E. coli VS181	Derivative of RP437; Δ(*cheYcheZ*)Δ*aer*Δ*tsr*Δ(*tar-tap*) Δ*trg*	(4)
E. coli CC118λpir	*araD* Δ(*ara*, *leu*) Δ*lacZ74 phoA20 galK thi-1 rspE rpoB argE recA1 λpir*	(5)
Pseudomonas aeruginosa PAO1 (WT-Hiroshima)	Prototroph, FP− (sex factor minus)	(6)
tlpQ::Km	PAO1 derivative, *PA2654*::Km; Km^R	(8)
pctA::Km	PAO1 derivative, *PA4309*::Km; Km^R	(9)
pctB::Km	PAO1 derivative, *PA4310*::Km; Km^R	(10)
pctC::Km	PAO1 derivative, *PA4307*::Km; Km^R	(10)
Pseudomonas aeruginosa PAO1 (WT-Washington)	Wild type strain	(7)
PA1251::ISlacZ	PAO1 derivative, *PA1251::ISlacZ*; Tc^R	(7)
PA1646::ISphoA	PAO1 derivative, *PA1646::ISphoA*; Tc^R	(7)
PA2867::ISlacZ	PAO1 derivative, *PA2867::ISlacZ*; Tc^R	(7)
PA2920::ISlacZ	PAO1 derivative, *PA2961::ISlacZ*; Tc^R	(7)
PA4290::ISphoA	PAO1 derivative, *PA4290::ISphoA*; Tc^R	(7)
PA4520::ISphoA	PAO1 derivative, *PA4520::ISphoA*; Tc^R	(7)

PA4915::ISphoA	PAO1 derivative, *PA4915::ISphoA*; Tc^R	(7)
mcpA::ISphoA	PAO1 derivative, *PA0180::ISphoA*; Tc^R	(7)
mcpS::ISlacZ	PAO1 derivative, *PA1930::ISlacZ*; Tc^R	(7)
ctpH::ISlacZ	PAO1 derivative, *PA2561::ISlacZ*; Tc^R	(7)
ctpM::ISlacZ	PAO1 derivative, *PA2652::ISlacZ*; Tc^R	(7)
mcpN::ISlacZ	PAO1 derivative, *PA2788::ISlacZ*; Tc^R	(7)
pctD::ISlacZ	PAO1 derivative, *PA4633::ISlacZ*; Tc^R	(7)
ctpL::ISphoA	PAO1 derivative, *PA4844::ISphoA*; Tc^R	(7)
Δ*McpK*	PAO1 derivative, Δ*PA5072*	(11)
pctP::Sm	PAO1 derivative, *PA1608*::Sm; Sm^R	This study

Plasmids

pET28a (+)	Km^R; Protein expression vector	Novagen
pKG116	Cm^R; Expression vector, salicylate inducible; for generation Type 2 and Type 3 hybrid chemoreceptor	(12)
pKNG101	Sm^R; OriR6K mob, mutant generation vector	(13)
pGEMT®	Ap^R; mutant generation vector	Invitrogen
pHC8	Ap^R; Expression vector, salicylate inducible; for generation type 1 hybrid chemoreceptor	This study
pSB13	Cm^R; Tar expression plasmid, pKG116 derivative, T768 was mutated to C768 to remove the NdeI restriction site	(14)
pVS88	Ap^R; CheY-EYFP / CheZ-ECFP expression plasmid used for type 2 and type 3 hybrid chemoreceptor	(4)
pWX16	Cm^R; CheY-EYFP / CheZ-ECFP expression plasmid used for type 1 hybrid chemoreceptor	This study
pOB30	Ap^R; GFP expression plasmid	(15)

pET28_PA2654-LBD	Km^R; pET28a (+) derivative containing a DNA fragment encoding PA2654-LBD	This study
pET28_PA4915-LBD	Km^R; pET28a (+) derivative containing a DNA fragment encoding PA4915-LBD	This study
pET28_PA4844-LBD	Km^R; pET28a (+) derivative containing a DNA fragment encoding PA4844-LBD	This study
pET28_PA5072-LBD	Km^R; pET28a (+) derivative containing a DNA fragment encoding PA5072-LBD	This study
pET28_PA2788-LBD	Km^R; pET28a (+) derivative containing a DNA fragment encoding PA2788-LBD	This study
pET28_PA4310-LBD	Km^R; pET28a (+) derivative containing a DNA fragment encoding PA4310-LBD	This study
pET28_PA4309-LBD	Km^R; pET28a (+) derivative containing a DNA fragment encoding PA4309-LBD	This study
pET28_PA4307-LBD	Km^R; pET28a (+) derivative containing a DNA fragment encoding PA4307-LBD	This study
pET28_PA4633-LBD	Km^R; pET28a (+) derivative containing a DNA fragment encoding PA4633-LBD	This study
pET28_PA1608-LBD	Km^R; pET28a (+) derivative containing a DNA fragment encoding PA1608-LBD	This study
pET28_PA2573-LBD	Km^R; pET28a (+) derivative containing a DNA fragment encoding PA2573-LBD	This study
pET28_PA2652-LBD	Km^R; pET28a (+) derivative containing a DNA fragment encoding PA2652-LBD	This study
pET28_PA2867-LBD	Km^R; pET28a (+) derivative containing a DNA fragment encoding PA2867-LBD	This study
pET28_PA2920-LBD	Km^R; pET28a (+) derivative containing a DNA fragment encoding PA2920-LBD	This study

pET28_PA1251-LBD	Km^R; pET28a (+) derivative containing a DNA fragment encoding PA1251-LBD	This study
pET28_PA1646-LBD	Km^R; pET28a (+) derivative containing a DNA fragment encoding PA1646-LBD	This study
pET28_PA2561-LBD	Km^R; pET28a (+) derivative containing a DNA fragment encoding PA2561-LBD	This study
pET28_PA4520-LBD	Km^R; pET28a (+) derivative containing a DNA fragment encoding PA4520-LBD	This study
PA2654-Tar_T1	Ap^R; pHC8 derivative containing a DNA fragment encoding PA2654 [1-373]-Tar [198-553];	This study
PA4915-Tar_T1	Ap^R; pHC8 derivative containing a DNA fragment encoding PA4915 [1-198]-Tar [198-553];	This study
PA4844-Tar_T1	Ap^R; pHC8 derivative containing a DNA fragment encoding PA4844 [1-312]-Tar [198-553];	This study
PA2573-Tar_T1	Ap^R; pHC8 derivative containing a DNA fragment encoding PA2573 [1-190]-Tar [198-553];	This study
PA2788-Tar_T1	Ap^R; pHC8 derivative containing a DNA fragment encoding PA2788 [1-187]-Tar [198-553];	This study
PA4310-Tar_T1	Ap^R; pHC8 derivative containing a DNA fragment encoding PA4310 [1-288]-Tar [198-553];	This study
PA4310-Tar_T2	Cm^R; pKG116 derivative containing a DNA fragment encoding PA4310 [1-353]-Tar [268-553];	(16)
PA4309-Tar_T1	Ap^R; pHC8 derivative containing a DNA fragment encoding PA4309 [1-288]-Tar [198-553];	This study
PA4309-Tar_T2	Cm^R; pKG116 derivative containing a DNA fragment encoding PA4309 [1-354]-Tar [269-553];	(16)
PA4307-Tar_T1	Ap^R; pHC8 derivative containing a DNA fragment encoding PA4307 [1-291]-Tar [198-553];	This study

PA4307-Tar_T2	Cm^R; pKG116 derivative containing a DNA fragment encoding PA4307 [1-356]-Tar [268-553];	(17)
PA4633-Tar_T1	Ap^R; pHC8 derivative containing a DNA fragment encoding PA4633 [1-369]-Tar [198-553];	This study
PA4633-Tar_T2	Cm^R; pKG116 derivative containing a DNA fragment encoding PA4633 [1-435]-Tar [267-553];	(18)
PA1608-Tar_T1	Ap^R; pHC8 derivative containing a DNA fragment encoding PA1608 [1-187]-Tar [198-553];	This study
PA1608-Tar_T2	Cm^R; pKG116 derivative containing a DNA fragment encoding PA1608 [1-264]-Tar [267-553];	This study
PA2652-Tar_T1	Ap^R; pHC8 derivative containing a DNA fragment encoding PA2652 [1-220]-Tar [198-553];	This study
PA2652-Tar_T2	Cm^R; pKG116 derivative containing a DNA fragment encoding PA2652 [1-284]-Tar [267-553];	This study
PA5072-Tar_T1	Ap^R; pHC8 derivative containing a DNA fragment encoding PA5072 [1-299]-Tar [198-553];	This study
PA5072-Tar_T3	Cm^R; pKG116 derivative containing a DNA fragment encoding PA5072 [1-299]-VLYHC-Tar [203-553];	This study
PA2867-Tar_T1	Ap^R; pHC8 derivative containing a DNA fragment encoding PA2867 [1-149]-Tar [198-553];	This study
PA2867-Tar_T3	Cm^R; pKG116 derivative containing a DNA fragment encoding PA2867 [1-149]-PTTTA-Tar [203-553];	This study
PA2920-Tar_T1	Ap^R; pHC8 derivative containing a DNA fragment encoding PA2920 [1-203]-Tar [198-553];	This study
PA2920-Tar_T3	Cm^R; pKG116 derivative containing a DNA fragment encoding PA2920 [1-203]-CLAPP-Tar [203-553];	This study
PA1251-Tar_T1	Ap^R; pHC8 derivative containing a DNA fragment encoding PA1251 [1-197]-Tar [198-553];	This study

PA1251-Tar_T3	Cm^{R}; pKG116 derivative containing a DNA fragment encoding PA1251 [1-197]-IWSLA-Tar [203-553];	This study
PA1646-Tar_T1	Ap^{R}; pHC8 derivative containing a DNA fragment encoding PA1646 [1-313]-Tar [198-553];	This study
PA1646-Tar_T3	Cm^{R}; pKG116 derivative containing a DNA fragment encoding PA1646 [1-313]-CLYMA-Tar [203-553];	This study
PA2561-Tar_T1	Ap^{R}; pHC8 derivative containing a DNA fragment encoding PA2561 [1-224]-Tar [198-553];	This study
PA2561-Tar_T3	Cm^{R}; pKG116 derivative containing a DNA fragment encoding PA2561 [1-224]-NYKNG-Tar [203-553];	This study
PA4520-Tar_T1	Ap^{R}; pHC8 derivative containing a DNA fragment encoding PA4520 [1-331]-Tar [198-553];	This study
PA4520-Tar_T3	Cm^{R}; pKG116 derivative containing a DNA fragment encoding PA2520 [1-331]-LICDS-Tar [203-553];	This study
pGEMT®-PA1608	Ap^{R}; pGEMT® derivative containing DNA fragment of PA1608	This study
pKNG101-PA1608	Sm^{R}, pKNG101 derivative containing DNA fragment of PA1608	This study

[a]Ap, ampicillin; Km, kanamycin; Tc, tetracycline; Cm, chloramphenicol; Sm, streptomycin

**Oligonucleotide**	**Sequence (5'-3')**	**Purpose**
PA2654-LBD_F	GTGCCGCGCGGCAGCCATATGCAACATTCCAGTGTTCTGGTTAAG	Construction of pET28_PA2654-LBD
PA2654-LBD_R	ACGGAGCTCGAATTCGGATCCCTATTCGCGGCGCATGTCATCAAGT	
PA4915-LBD_F	GTGCCGCGCGGCAGCCATATGTCTCGTCTGGACGCAAGCTT	Construction of pET28_PA4915-LBD
PA4915-LBD_R	ACGGAGCTCGAATTCGGATCCCTAGCGCATACTATCGTAGCGTTT	

PA4844-LBD_F	GTGCCGCGCGGCAGCCATATGCGCGCCATGGAACGTCCGT	Construction of pET28_PA4844-LBD
PA4844-LBD_R	ACGGAGCTCGAATTCGGATCCCTACTGCCCTTGCAAACGCTGAC	
PA2573-LBD_F	GTGCCGCGCGGCAGCCATATGCGCCTTGGGAAAGCATTAAGTG	Construction of pET28_PA2573-LBD
PA2573-LBD_R	ACGGAGCTCGAATTCGGATCCCTAACGCATGCTATCTTCACGCGC	
PA2788-LBD_F	GTGCCGCGCGGCAGCCATATGAGTATGAGCATCTCGCCGGAAA	Construction of pET28_PA2788-LBD
PA2788-LBD_R	ACGGAGCTCGAATTCGGATCCCTACTGAGTATGCTGAACAGAATCC	
PA4310-LBD_F	GTGCCGCGCGGCAGCCATATGAATGATAGCTTACAGCGTGCTT	Construction of pET28_PA4310-LBD
PA4310-LBD_R	ACGGAGCTCGAATTCGGATCCCTAGCTAGTGCGCAGTTTGGTT	
PA4309-LBD_F	GTGCCGCGCGGCAGCCATATGAACGACTACTTACAGCGCAAC	Construction of pET28_PA4309-LBD
PA4309-LBD_R	ACGGAGCTCGAATTCGGATCCCTAGCTAACGCGAAATTTTGACAGC	
PA4307-LBD_F	GTGCCGCGCGGCAGCCATATGGACTACCGTCAGCGTGAAG	Construction of pET28_PA4307-LBD
PA4307-LBD_R	ACGGAGCTCGAATTCGGATCCCTACGAGGTACGAAACTCTGAG	
PA4633-LBD_F	GTGCCGCGCGGCAGCCATATGCGCACCCAAGAACTGGTTC	Construction of pET28_PA4633-LBD
PA4633-LBD_R	ACGGAGCTCGAATTCGGATCCCTAACTCATCCCCAGAATATCCTG	
PA1608-LBD_F	GTGCCGCGCGGCAGCCATATGCGCATGGGCAGCCTGAACGA	Construction of pET28_PA1608-LBD
PA1608-LBD_R	ACGGAGCTCGAATTCGGATCCCTAGCTGTGGTATACTTCATCA	
PA2652-LBD_F	GTGCCGCGCGGCAGCCATATGAAGAAGCAGGCCGATGCCGA	Construction of pET28_PA2652-LBD
PA2652-LBD_R	ACGGAGCTCGAATTCGGATCCCTAGGTGCCGATGCGCTCGTCAAT	

PA5072-LBD_F	GTGCCGCGCGGCAGCCATATGAGCAATCGTACCTTAACCCATC	Construction of pET28_PA5072-LBD
PA5072-LBD_R	ACGGAGCTCGAATTCGGATCCCTAACGATCACGGGACGCGGTA	
PA2867-LBD_F	GTGCCGCGCGGCAGCCATATGCAACAAGAAGCCGCGGGG	Construction of pET28_PA2867-LBD
PA2867-LBD_R	ACGGAGCTCGAATTCGGATCCCTAACGATCTGCAAACACTTGCCAA	
PA2920-LBD_F	GTGCCGCGCGGCAGCCATATGCAATTAAATGAAAGCAATCAACG	Construction of pET28_PA2920-LBD
PA2920-LBD_R	ACGGAGCTCGAATTCGGATCCCTACTGCTGCCAACGATGGC	
PA1251-LBD_F	GTGCCGCGCGGCAGCCATATGCGCATGGGTAGCCTGAATCG	Construction of pET28_PA1251-LBD
PA1251-LBD_R	ACGGAGCTCGAATTCGGATCCCTAATCTGCCGCCTCAGCTGTAGA	
PA1646-LBD_F	GTGCCGCGCGGCAGCCATATGCAACGCTTTGCCCGCTTACA	Construction of pET28_PA1646-LBD
PA1646-LBD_R	ACGGAGCTCGAATTCGGATCCCTACGAATTTGTGCGCAGGGAGTG	
PA2561-LBD_F	GTGCCGCGCGGCAGCCATATGACCGGTGATGTGCGTGCATAT	Construction of pET28_PA2561-LBD
PA2561-LBD_R	ACGGAGCTCGAATTCGGATCCCTAGGTGCGACGCGCCTCAGCGCT	
PA4520-LBD_F	GTGCCGCGCGGCAGCCATATGTATCTGGTACGTGACGCATAC	Construction of pET28_PA4520-LBD
PA4520-LBD_R	ACGGAGCTCGAATTCGGATCCCTACTGCGTACGGACCTGTTGT	
PA1608_MUT_F	AGTTTCGCCCTGATCACCG	Construction of pKNG101-PA1608
PA1608_MUT_R	TTCCTGGATGTTGGCGATCAT	
pKG116-seq_F	AAGCCATAAGGAGTACCATATG	Sequencing for hybrid Chemoreceptor_T2&T3
pKG116-seq_R	TTACTTATTTATCCGCGGATC	
pET28a-seq_F	GTGCCGCGCGGCAGCCATATG	Sequencing for LBD expression plasmids
pET28a-seq_R	ACGGAGCTCGAATTCGGATCCCTA	
Hybrid-Seq_F	AGCCATAAGGAGTACCATATG	Sequencing for hybrid

Hybrid-Seq_R	AGCAGAATCAATACCACCAC	Chemoreceptor_T1
PA1608-T2_F1	AGCCATAAGGAGTACCATATGATGTCTTTGCGCAGT ATGCC	Construction of PA1608-Tar_T2
PA1608-T2_R1	CCTTCGCGGACATGCTGGATGGTCTGGCGCAGTTG	
PA1608-T2_F2	CATCCAGCATGTCCGCGAAGGTTCAGAT	
Tar-T2_R2	GGATCCTCAAAATGTTTCCCAGTTT	
PA2652-T2_F1	AGCCATAAGGAGTACCATATGATGCGTCTGACCCTG AA	Construction of PA2652-Tar_T2
PA2652-T2_R1	CCTTCGCGGACATGCCGCACCAGACCGTGGATC	
PA2652-T2_F2	GTGCGGCATGTCCGCGAAGGTTCAGAT	
Tar-T3_F	TTGATTCTGCTGGTGGCGTG	Construction for hybrid Chemoreceptor_T3
pKG116_Tar-T3_R	TACTTATTTATCCGCGGATCCTCAAAATGTTTCCCAG TTTGGATCTTG	
PA5072-T3_F	AGCCATAAGGAGTACCATATGATGTATGATTGGTGG GTCTTG	Construction of PA5072-Tar_T3
PA5072-T3_R	CCACCAGCAGAATCAANNNNNNNNNNNNNNNGGCT GCAATCAGCCATAA	
PA2867-T3_F	AGCCATAAGGAGTACCATATGATGGGTACGTGGATT TCGGAT	Construction of PA2867-Tar_T3
PA2867-T3_R	CCACCAGCAGAATCAANNNNNNNNNNNNNNNAAGC ATCAGCACTAAAAC	
PA2920-T3_F	AGCCATAAGGAGTACCATATGATGCTGCAATGGTTC GCC	Construction of PA2920-Tar_T3
PA2920-T3_R	CCACCAGCAGAATCAANNNNNNNNNNNNNNNCAAC AGTCCAAAGGCAAC	
PA1251-V3_F	AGCCATAAGGAGTACCATATGATGCTTCTTCGTCGCA TT	Construction of PA1251-Tar_T3
PA1251-T3_R	CCACCAGCAGAATCAANNNNNNNNNNNNNNNTAAC AACACTGCCAGAATG	
PA1646-T3_F	AGCCATAAGGAGTACCATATGATGTTGGGATTATTA CGTCG	Construction of PA1646-Tar_T3
PA1646-T3_R	CCACCAGCAGAATCAANNNNNNNNNNNNNNNAACC AGCAATGCTAAAAC	
PA2561-T3_F	AGCCATAAGGAGTACCATATGATGCCTGCTTCTCCTG G	Construction of PA2561-Tar_T3

PA2561-T3_R	CCACCAGCAGAATCAANNNNNNNNNNNNNNNNACCAATCAGCACCAATGA	
PA4520-T3_F	AGCCATAAGGAGTACCATATGATGAAAACCGTATTGTACCCG	Construction of PA4520-Tar_T3
PA4520-T3_R	CCACCAGCAGAATCAANNNNNNNNNNNNNNNNCACCACCGCCAACGCTGC	

Table S4 Experimental conditions used for microcalorimetric titrations

Protein		Ligand		Temperature (ºC)	Injection volume (µL)	Analysis buffer composition
Name	Concentration (µM)	Name	Concentration (mM)			
PctA-LBD	39	L-ornithine	1	30	4.8	5 mM Tris, 5 mM Pipes, 5 mM Mes, 10 % glycerol (vol/vol), 150 mM NaCl, pH 7.0
PctB-LBD	50	L-ornithine	20	30	12.8	5 mM Tris, 5 mM Pipes, 5 mM Mes, 10 % glycerol (vol/vol), 150 mM NaCl, pH 7.0
PctC-LBD	120	Methyl 4-aminobutyrate	20	25	14.4	5 mM Tris, 5 mM Pipes, 5 mM Mes, 10 % glycerol (vol/vol), 150 mM NaCl, pH 7.0
	120	5-aminovalerate	5	25	3.2	
TlpQ-LBD	93	2-phenylethylamine	20	15	11.2	5 mM Tris, 5 mM Pipes, 5 mM Mes, 10 % glycerol (vol/vol), 150 mM NaCl, pH 7.0
	147	Tyramine	20	25	6.4	
PA1608-LBD	65	Hypoxanthine	2	20	4.8	10 mM sodium phosphate, pH 5.5
PA4915-LBD	92	2-phenylethylamine	5	25	12.8	40 mM Potassium phosphate, 10 % glycerol (vol/vol), pH 7.0

References

1. Jeong H, Barbe V, Lee CH, Vallenet D, Yu DS, Choi SH, Couloux A, Lee SW, Yoon SH, Cattolico L, Hur CG, Park HS, Ségurens B, Kim SC, Oh TK, Lenski RE, Studier FW, Daegelen P, Kim JF. 2009. Genome sequences of *Escherichia coli* B strains REL606 and BL21(DE3). J Mol Biol 394:644–652.

2. Woodcock DM, Crowther PJ, Doherty J, Jefferson S, Decruz E, Noyer-Weidner M, Smith SS, Michael MZ, Graham MW. 1989. Quantitative evaluation of *Escherichia coli* host strains for tolerance to cytosine methylation in plasmid and phage recombinants. Nucleic Acids Res 17:3469–3478.

3. Ames P, Studdert CA, Reiser RH, Parkinson JS. 2002. Collaborative signaling by mixed chemoreceptor teams in *Escherichia coli*. Proc Natl Acad Sci U S A 99:7060–7065.

4. Sourjik V, Berg HC. 2004. Functional interactions between receptors in bacterial chemotaxis. Nature 428:437–441.

5. Herrero M, De Lorenzo V, Timmis KN. 1990. Transposon vectors containing non-antibiotic resistance selection markers for cloning and stable chromosomal insertion of foreign genes in gram-negative bacteria. J Bacteriol 172:6557–6567.

6. Holloway BW, Krishnapillai V, Morgan AF. 1979. Chromosomal genetics of *Pseudomonas*. Microbiol Rev 43:73–102.

7. Jacobs MA, Alwood A, Thaipisuttikul I, Spencer D, Haugen E, Ernst S, Will O, Kaul R, Raymond C, Levy R, Chun-Rong L, Guenthner D, Bovee D, Olson M V., Manoil C. 2003. Comprehensive transposon mutant library of *Pseudomonas aeruginosa*. Proc Natl Acad Sci U S A 100:14339–14344.

8. Kim HE, Shitashiro M, Kuroda A, Takiguchi N, Kato J. 2007. Ethylene Chemotaxis in *Pseudomonas aeruginosa* and Other *Pseudomonas* Species. Microbes Environ 22:186–189.

9. Kuroda A, Kumano T, Taguchi K, Nikata T, Kato J, Ohtake H. 1995. Molecular cloning and characterization of a chemotactic transducer gene in *Pseudomonas aeruginosa*. J Bacteriol 177:7019.

10. Taguchi K, Fukutomi H, Kuroda A, Kato J, Ohtake H. 1997. Genetic identification of chemotactic transducers for amino acids in *Pseudomonas aeruginosa* 3223–3229.

11. Martín-Mora D, Ortega A, Reyes-Darias JA, García V, López-Farfán D, Matilla MA, Krell T. 2016. Identification of a Chemoreceptor in *Pseudomonas aeruginosa* That Specifically Mediates Chemotaxis Toward α-Ketoglutarate. Front Microbiol 7.

12. Burón-Barral MDC, Gosink KK, Parkinson JS. 2006. Loss- and gain-of-function mutations in the F1-HAMP region of the *Escherichia coli* aerotaxis transducer Aer. J Bacteriol 188:3477–3486.

13. Kaniga K, Delor I, Cornelis GR. 1991. A wide-host-range suicide vector for improving reverse genetics in Gram-negative bacteria: inactivation of the blaA gene of *Yersinia enterocolitica*. Gene 109:137–141.

14. Bi S, Jin F, Sourjik V. 2018. Inverted signaling by bacterial chemotaxis receptors. Nat Commun 9.

15. Bi S, Pollard AM, Yang Y, Jin F, Sourjik V. 2016. Engineering Hybrid Chemotaxis Receptors in Bacteria. ACS Synth Biol 5:989–1001.

16. Reyes-Darias JA, Yang Y, Sourjik V, Krell T. 2015. Correlation between signal input and output in PctA and PctB amino acid chemoreceptor of *Pseudomonas aeruginosa*. Mol Microbiol 96:513–525.

17. Reyes-Darias JA, García V, Rico-Jiménez M, Corral-Lugo A, Lesouhaitier O, Juárez-Hernández D, Yang Y, Bi S, Feuilloley M, Muñoz-Rojas J, Sourjik V, Krell T. 2015. Specific gamma-aminobutyrate chemotaxis in *pseudomonads* with different lifestyle. Mol Microbiol 97:488–501.

18. Matilla MA, Velando F, Tajuelo A, Martín-Mora D, Xu W, Sourjik V, Gavira JA, Krell T. 2022. Chemotaxis of the Human Pathogen *Pseudomonas aeruginosa* to the Neurotransmitter Acetylcholine. MBio 13.
